# Exploratory study linking plasma proteomics to cardiotoxicity in Hodgkin lymphoma

**DOI:** 10.1186/s40959-025-00426-2

**Published:** 2025-12-26

**Authors:** Johan Mattsson Ulfstedt, Ragnhild Risebro, Eva Freyhult, Christina Christersson, Charlott Mörth, Masood Kamali-Moghaddam, Anna Robelius, Gunilla Enblad, Daniel Molin

**Affiliations:** 1 Department of Immunology, Genetics & Pathology, Cancer Immunotherapy, Uppsala University, Uppsala, Sweden; 2 Department of Cell and Molecular Biology, Science for Life Laboratory, National Bioinformatics Infrastructure Sweden, Uppsala University, Uppsala, Sweden; 3 Department of Medical Sciences, Uppsala University, Uppsala, Sweden; 4 Centre for Clinical Research at Uppsala University, Mälarsjukhuset, Eskiltuna, Sweden; 5 Department of Immunology, Genetics & Pathology, Science for Life Laboratory, Uppsala University, Uppsala, Sweden

**Keywords:** Proteomics, Classical hodgkin lymphoma, Cardiac toxicity, Doxorubicin, Radiation therapy

## Abstract

**Background:**

Cardiovascular toxicity is a well-known complication of chemotherapy, especially doxorubicin (DXR), and irradiation of the mediastinum for classical Hodgkin lymphoma (cHL). Due to the excellent prognosis in cHL, the mortality rate in late toxicity historically exceeds that of relapse of lymphoma. This highlights the need for strategies to minimize toxicity.Our aim was to characterize the prevalence of cardiovascular diseases (CVDs) in our cohort of cHL patients treated with DXR with or without radiotherapy according to standard practice and to identify any plasma protein associations with preexisting or emerging CVD posttreatment.

**Methods:**

We analyzed 182 different proteins in plasma samples from 56 cHL patients and 60 controls using Olink multiplex protein panels Oncology II and Cardiovascular III. The analysis was supplemented with separate analyses of N-terminal pro-brain natriuretic peptide (NTpro-BNP), troponin I and C-reactive protein (CRP). The patient samples were prospectively collected prior to, during and after treatment.

**Results:**

Our analysis revealed a statistically significant association between the compound endpoint of heart failure and ischemic heart disease and the protein biomarkers cysteine rich protein 61 (CYR61), glycoprotein nonmetastatic melanoma protein B (GPNMB) and activated leukocyte cell adhesion molecule (ALCAM) in samples collected after treatment for cHL.

**Conclusion:**

This exploratory study identified three new biomarkers reflecting different biological processes associated with CVD in patients treated for cHL. Adding biomarkers to risk prediction in this population has the potential to identify patients with a high risk of cardiovascular events who need focused follow-up.

**Supplementary Information:**

The online version contains supplementary material available at 10.1186/s40959-025-00426-2.

## Introduction

The prognosis for patients diagnosed with classical Hodgkin lymphoma (cHL) is generally excellent, with more than 90% 5-year disease-free survival [1]. In younger patients and early stages, for which the prognosis in cHL is most favorable, the mortality of late treatment effects historically exceeds that of cHL 15–20 years after treatment [2, 3]. Non-relapse mortality in this group of young patients is predominantly associated with cardiovascular diseases (CVDs) and secondary malignancies. The cardiotoxicity of cHL treatment can be divided into indirect and direct cardiotoxic effects. The indirect effects are driven by an increased prevalence of known risk factors for CVD, where survivors of cancer among adolescents and young adults have shown an increased incidence of diabetes mellitus (DM), hyperlipidemia and hypertension (HT) [4]. The direct toxicity is driven by doxorubicin (DXR), an anthracycline that is associated with a drug class dose-limiting cardiac toxicity, which manifests as ischemia, arrythmia, left ventricular dysfunction and heart failure (HF) [5–7]. The damage may present during the treatment, but also decades after treatment completion. The cardiotoxicity of anthracyclines in cHL is further aggravated by radiation therapy that, due to the distribution of the disease in cHL, often involves the mediastinum. The left anterior descending artery (LAD) is of specific concern due to the risk of radiation-induced acceleration of the atherosclerotic process. Moreover, valvular disease, pericardial disease and left ventricular function are also important considerations, especially in cHL cases, where a large part of the heart volume is irradiated [8–10]. Elderly patients with preexisting cardiovascular comorbidities are particularly vulnerable in this regard and have been shown to have poorer prognosis in cHL [11].

There is evidence for both direct cardiotoxic effects associated with DXR in a dose-dependent manner and indirect cardiotoxic effects of chemotherapy in general, leading to an increased prevalence of CVDs and lower quality of life for survivors of cHL. This highlights the need for early markers of CVD in survivors of cHL, as also recently pointed out in an editorial in the Journal of the American College of Cardiology [12, 13].

We hypothesize that biomarkers associated with CVD could be used to individualize the treatment strategy based on an individual cardiovascular morbidity risk profile, improving overall survival for both younger and elderly patients with cHL. In this exploratory study, we measured the plasma protein profile of 58 cHL patients using Olink™ Proteomics. Samples were analyzed at baseline, during treatment and during follow-up. As a reference, 60 samples from healthy controls were used.

## Materials and methods

Fifty-six patients with primary (*n* = 54) or recurrent (*n* = 2) cHL were recruited as part of the Uppsala Umeå Comprehensive Cancer Consortium (U-CAN) project [14] between September 2010 and November 2016 (patient characteristics shown in Table 1). The patients were included from a real-world single center university hospital setting, where patients were treated according to standard treatment protocols or included in clinical trials. Due to logistical reasons and changes in routine sampling points over time, not all patients have consistent biobank coverage. EDTA plasma samples were released from the U-CAN biobank for the plasma proteome analysis under ethical approval (dnr. 2013/059 and dnr. 2014/233). For comparison, the biobanked plasma proteome of 60 controls without known malignancies (30 male, 30 nonpregnant females) recruited as part of the EpiHealth survey [15] was analyzed, and their clinical characteristics were retrieved from questionnaires. Data collection in the EpiHealth study and usage of the material in this project was approved by the Ethics Committee of Uppsala (dnr. 2010/402: 2010-12−01, 2011-11−17, dnr. 2015/179). The EpiHealth study is approved by the Swedish Data Protection Authority.

**Table 1:** Characteristics of classical Hodgkin lymphoma patients and controls

Variable	**cHL**n = 56		**Controls**n = 60
	At diagnosisMean (SD)or n (%)	At follow upMean (SD)or n (%)	Mean (SD)or n (%)
Age	46.2 (19.4)	53.8 (18.6)	67.0 (8.4)
Sex			
Female	21 (37.5)		30 (50)
Male	35 (62.5)		30 (50)
Risk factors for CVD			
BMI	25.9 (4.7)	NA	25.0 (3.9)
Diabetes mellitus	3 (5.3)	4 (7.1)	2 (3.3)
Hypertension	13 (23.0)	22 (37.9)	14 (23.3)
CVD	7 (13.0)	14 (25.9)	3 (5.0)
Atrial fibrillation	4 (6.7)	5 (8.6)	0 (0)
Pacemaker	1 (1.7)	1 (1.7)	NA
Heart failure	1 (1.7)	6 (11.0)	0 (0)
Coronary artery disease	3 (5.2)	4 (6.9)	2 (3.3)
Thrombosis	2 (3.4)	9 (15.5)	NA
DVT PE	2 (3.4)0 (0)	8 (13.8) 2 (3.4)	NANA
Vascular disease	4 (6.9)	5 (8.6)	1 (1.7)
TIA	2 (3.7)	3 (5.2)	NA
Stroke	2 (3.7)	2 (3.7)	NA
Peripheral arterial disease	0 (0)	0 (0)	NA

Clinical data for the 56 cHL patients included in the statistical analysis were collected from an electronic patient charting system and included age at diagnosis, date of diagnosis, sex, international prognostic score (IPS), DXR dose, body mass index (BMI), body surface area (BSA), estimated glomerular filtration rate (eGFR estimated according to the Lund-Malmö model) [16, 17], C-reactive protein (CRP), DM, HT, venous thrombosis and current medications. Due to the generally excellent prognosis in cHL, this study was not powered to show any differences in survival.

A review of medical records was conducted to evaluate the presence of CVD at diagnosis and the development of additional CVD events after the initiation of lymphoma treatment. Cardiac disease was classified as HF, coronary artery disease, coronary artery intervention (percutaneous intervention or coronary bypass), cardiac arrest, atrial fibrillation, presence of a pacemaker, or clinically relevant valvular disease. Vascular disease was defined as peripheral arterial disease, carotid stenosis, aortic aneurysm, deep vein thrombosis (DVT), pulmonary embolism (PE), arterial thrombosis/ischemic stroke, or transient ischemic attack (TIA). The presence of risk factors such as DM and HT at diagnosis or subsequent development of such risk factors posttreatment were also noted.

The Olink™ multiplex protein extension assay (PEA) panels CVD III/ONC II (Olink™ Proteomics, Uppsala Sweden) were used in our study. Each panel allows for the simultaneous analysis of 92 individual plasma proteins. Thirty-seven samples were collected at diagnosis, 20 samples after two cycles of treatment, 20 samples at follow-up, 4 samples at relapse and 1 sample after relapse treatment. Follow-up samples were collected on average 4 months post treatment (range 1 month to 14 months). The assay is designed for analysis of limited quantities of sample, where each plasma protein analysis is performed using a sample volume of only 1 µL. The CVD III panel contains a prespecified fixed set of 92 known and prospective biomarkers associated with inflammation and CVD, whereas the ONC II panel consists of a prespecified fixed set of cancer-related proteins associated with cancer initiation and progression, including biomarkers for angiogenesis and inflammation. The PEA technique is based on dual antibody-DNA probes that bind to the same protein, ensuring high specificity. Upon binding, the DNA strands hybridize and are extended to form templates for qPCR, generating a signal proportional to protein concentration. Results are reported as normalized protein expression (NPX), with controls used to determine detection limits and normalize the data [18].

The N-terminal pro-brain natriuretic peptide (NTpro-BNP) and CCL22 analyses in the CVD III panel did not meet the minimum quality requirements specified by the manufacturer and were therefore excluded from the analysis. The PEA assay was complemented by a separate analysis of the key cardiovascular biomarkers troponin I and NT-proBNP using chemical luminescence methods on an Architect i2000SR from Abbot. CRP values were collected from patient records and analyzed according to local clinical routine at the time of diagnosis.

### Statistical analysis

Due to the size of the sample and limited follow-up time, a compound endpoint of CVD was studied. For the purpose of statistical robustness, a statistical analysis was only performed if a minimum of five new CVD events had accumulated at the analysis time point. Associations between protein levels before/after treatment and the compound endpoint of CVD were assessed using linear regression with adjustment for age at diagnosis, sex and BMI. Multiple testing correction was performed using Benjamini‒Hochberg´s false discovery rate method, adjusting for the number of investigated proteins (182). The adjusted p value, called q-value, is reported in the results. The significance threshold was set at q = 0.10.

## Results

### Patient population

The disease characteristics, treatments and risk factors are listed in Table 2. Chemotherapy doses were retrieved from medical records, and where the exact doses were not available, the doses were estimated based on the patient characteristics and dose reductions as specified in patient notes. Two patients, who did not receive any cardiotoxic treatment, were excluded from the statistical analysis, leaving in total 56 patients in the study. The mean DXR dose was 234 mg/m^2^ (SD = 81.02) over 0 to 8 cycles of treatment. The median follow-up in the cHL cohort was 6.5 years (SD = 4.9). Eleven patients received radiation therapy including the mediastinum as part of their treatment. Eight patients received 29.75 Gy, one patient received 20 Gy, one patient received 24 Gy and one patient received 45.4 Gy. Calculated heart doses and LAD doses were not available.

**Table 2:** Patient characteristics

Variable	cHL *n* = 56
	At diagnosis Mean (SD) or *n* (%)
Histology	
Nodular sclerosis	37 (66)
Mixed cellularity	5 (9)
Lymphocyte predominant	1 (2)
Lymphocyte depleted	0 (0)
cHL -NOS*	12 (21)
NA	1
Stage	
I	7 (13)
II	21 (38)
III	15 (26)
IV	13 (23)
Risk factors (stage IA-IIA)	23
0	7
1	12
2	4
IPS (stage IIB-IVB)	33
1	6
2	6
3	11
4	9
5	1
B-symptoms	
no	33 (59)
yes	23 (41)
Treatment	
ABVD	45 (80)
BEACOPP	2 (3)
CHOP variant	8 (14)
NA	1 (2)
Radiotherapy	24 (43)
Deaths	
Total	10 (17)
Treatment related	2 (3)
Disease related	4 (7)
Other	4 (7)
Hemoglobin (g/L)	129.2 (17.76)
Leukocyte count (x 10^9^/L)	10.02 (4.22)
Lymphocyte count (x 10^9^/L)	1.6 (0.57)
Lymphocyte count (%)	17.6 (8.64)
ESR (mm/h)	39.6 (33.65)
C-reactive protein (mg/L)	44.9 (48.00)
Albumin (g/L)	33.98 (6.07)
Lactate dehydrogenase (µkat/L)	3.4 (1.45)
eGFR (mL/min)	88.9 (18.62)
DXR dose (mg/m^2^)	234 (81.02)
BSA (m ^2^)	1.96 (0.22)

*Classical HL not otherwise specified histology (cHL-NOS)

**ICE, Bendamustin, Rituximab, CEOP

### Risk factors for cardiovascular disease

At diagnosis, the average BMI of the patients was 26.0 kg/m^2^ compared to 25.0 kg/m^2^ for the control group. There were 3 patients (5.6%) with DM in the cHL cohort at diagnosis and it increased to 4 patients (7.1%) during the follow-up period, while the prevalence of DM in the control group was 3.3%. There were 13 patients (23.2%) with HT in the cHL cohort at diagnosis which increased to 20 patients (35.7%) during the follow-up period. The prevalence in the control group was 23.3%. The mean age at diagnosis for the cHL cohort was 46.2 years and at follow-up 54.7 years, compared to the mean age in the control group of 67.0 years (*p* = 6.19⋅10^− 9^).

### Prevalence of cardiovascular diseases at diagnosis

At the time of diagnosis, 7 (12.5%) of the patients in the cHL cohort had preexisting CVD. Four (7.1%) patients had AF, 1 (1.8%) patient had preexisting HF, 3 (5.4%) had coronary artery disease, 4 (7.1%) had stroke/TIA and 2 (3.6%) had previous venous thrombosis.

### Prevalence of cardiovascular diseases at follow-up

At follow-up, 14 (25.0%) patients in the cHL cohort had developed CVD. Four (7.1%) patients had AF, 8 (14.2%) had HF, 5 (8.9%) had coronary artery disease, 5 (8.9%) had stroke/TIA, and 8 (14.3%) had developed venous thrombosis. Only one of these 14 patients had also a prior history of CVD, the remaining cases represented new-onset CVD.

In the reference cohort, 3 subjects (5%) had CVD at the time of inclusion. Two (3.3%) subjects had been diagnosed with coronary artery disease, one (1.7%) with vascular disease and none had AF, HF or peripheral arterial disease. A history of venous thrombosis was not included in the health questionnaire provided by the controls as part of the EpiHealth cohort. The characteristics of the cHL cohort and the control group are summarized in Table 1, and the disease characteristics and treatments in the cHL cohort are summarized in Table 2.

### Protein biomarker analysis

**Fig. 1:**
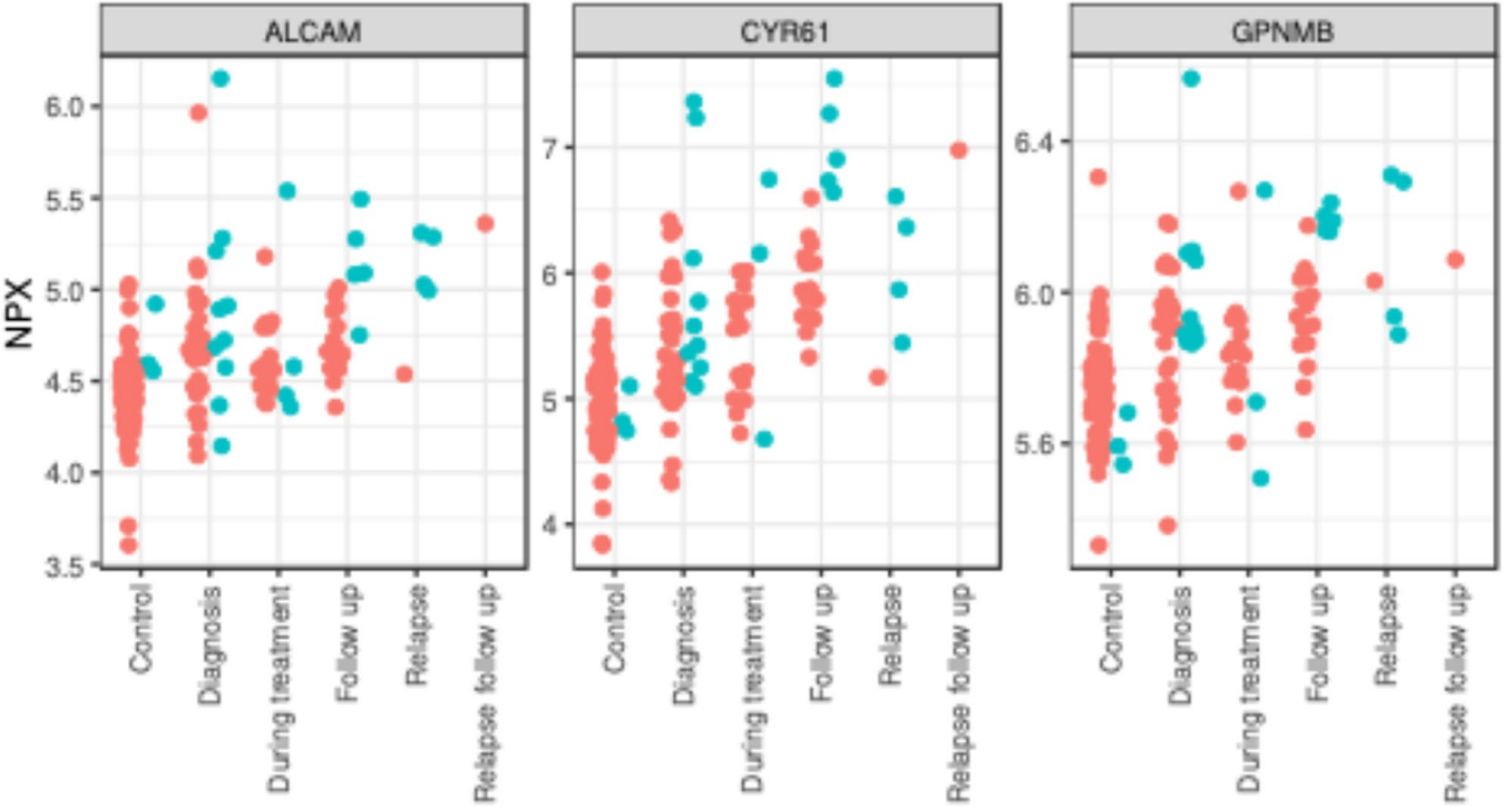
Distribution of NPX values for ALCAM, CYR61 and GPNMB. Comparison of the distribution of NPX values for the EpiHealth control cohort and cHL cohort. Patients with CVD events in green and individuals without CVD in red

In our analysis, none of the traditional biomarkers Troponin I, NT-proBNP or CRP [19, 20] for cardiovascular toxicity were associated with CVD in our cHL cohort at any sample point. In addition, we did not find any association between CVD and the protein biomarkers in samples collected at diagnosis or during treatment. We identified three proteins, CYR61 (log_2_FC = 1.13, *p* = 0.000047, q = 0.0086), GPNMB (log_2_FC = 0.29, *p* = 0.00078, q = 0.054) and ALCAM (log_2_FC = 0.48, *p* = 0.00088, q = 0.054), with significantly higher protein levels after treatment in samples from patients who developed CVD than in samples from patients without CVD. Figure 1 shows the distribution of PX values in the EpiHealth cohort compared to the cHL cohort. The results for all protein biomarkers are included in Supplemental Tables 1 and 2.

## Discussion

At diagnosis, we noted a high prevalence of CVD in the cHL cohort compared to the control group, despite being considerably younger both at the time of diagnosis and at follow-up. Schoormans et al. reported a lower incidence of CVD in cHL at diagnosis compared to other malignancies [21], and Fowler et al. found a lower prevalence of comorbidities among cHL patients compared to patients with colon, rectal, and lung cancers [22]. The absence of healthy control groups in both studies makes comparisons difficult, and Fowler et al. had insufficient numbers of CVD events for a comparative statistical analysis in their material. Our finding warrants further investigation but could be explained by a surveillance bias, as previously noted in chronic lymphatic leukemia [23]. After 7.8 years of follow-up, we noted a marked increase in HF and venous thrombosis as early signs of treatment-related cardiovascular toxicity in our cHL cohort. At longer follow-up, we would expect an accelerated atherosclerotic effect to begin to manifest in the form of coronary artery disease and valvular disease.

In our analysis, higher levels of cysteine-rich protein 61 (CYR61), glycoprotein nonmetastatic melanoma protein B (GPNMB) and activated leukocyte cell adhesion molecule (ALCAM) correlated with the development of CVD after treatment. Classical biomarkers for CVD, such as troponin I and NTpro-BNP, did not show any association with the CVD endpoint in this study. We believe that this is associated with the timing of sample collection, as follow-up samples were collected on average 3 months posttreatment. Our analysis thus reflects the acute phase of treatment-related side effects, while troponin I and NTpro-BNP reflect the chronic phase, which manifests years after treatment completion.

### Cysteine-rich protein 61 (CYR61)

The CYR61 protein, also referred to as cellular communication network factor 1 (CCN1), has four distinct domains: an insulin-like growth factor binding domain, a von Willebrand factor domain, a thrombospondin homology domain and a heparin binding domain [24, 25], suggesting a wide range of mechanisms of action associated with tissue damage repair and angiogenesis [26]. CYR61 is known to be associated with several solid tumors [25], where angiogenesis plays an important role [26]. To our knowledge, no association between CYR61 and cHL has previously been reported.

CYR61 has been found to be differentially expressed in tissues experiencing ischemia, such as stroke and coronary artery disease [27, 28] Patients diagnosed with acute coronary syndrome displayed higher serum CYR61 levels than patients with stable angina, although patients with stable angina had higher serum levels than healthy controls. CYR61 is also associated with atherosclerotic lesions [29] and associated with poor prognosis in acute coronary syndrome [30]. Elevated levels of serum CYR61 are also correlated with acute HF where higher levels correlate with poor prognosis [31, 32]. It is believed that CYR61 is associated with neovascularization and that the protein is induced as a part of the hypoxic tissue response through different signaling pathways, including vascular endothelial growth factor (VEGF) [33] and angiotensin II [29].

### Glycoprotein nonmetastatic melanoma protein B (GPNMB)

GPNMB, also known as hematopoietic growth factor inducible neurokinin type 1 (GHFIN) or dendritic cell heparan sulfate proteoglycan integrin dependent ligand (DCHIL), is a membrane bound protein constitutively expressed in most tissues, with increased expression in macrophages and microglia in response to proinflammatory stimuli [34]. The current body of evidence suggests that GPNMB has a predominantly anti-inflammatory role.

Little is known about the association between GPNMB and CVD in humans. Data from animal models suggest that GPNMB plays an important role in suppressing macrophage- and microglia-induced inflammatory responses [34]. Overweight GPNMB-knockout mice were found to have an increased risk of developing low-grade adipose tissue inflammation, insulin resistance and liver fibrosis associated with type 2 diabetes [35]. GPNMB knockout mice displayed resistance to cardiac remodeling associated with HF following myocardial infarction [36]. GPNMB also appears to have a neuroprotective role in mouse models of ischemic stroke [37]. A marked increase in GPNMB was noted in a rabbit model of atherosclerosis when the animals were fed a high cholesterol diet for five weeks [38]. A previously identified risk allele for Takayasu vasculitis appears to suppress the expression of GPNMB in cell cultures as a plausible mechanism for the underlying pathophysiology in this disease [39]. Thus, there seems to be evidence for the protective effect of GPNMB in relation to vascular damage associated with myocardial infarctions and HF.

### Activated leukocyte cell adhesion molecule (ALCAM)

ALCAM, also known as CD166, belongs to the immunoglobulin superfamily of proteins expressed on activated leucocytes [40]. ALCAM is also associated with embryological hematopoiesis and cardiovascular development [41, 42], as well as cancer progression and metastasis [43].

ALCAM is an adhesion molecule that is upregulated on activated monocytes and believed to be associated with monocyte migration [44]. It is associated with immune cell migration into the central nervous system in multiple sclerosis [45]. ALCAM expression also correlates with long-term mortality in ischemic stroke [46]. Elevated levels of serum ALCAM are associated with chronic inflammatory conditions such as chronic obstructive pulmonary disease and prognosis in lung cancer [47]. More recently, ALCAM has been connected to cardiovascular death following myocardial infarction [48].

It is plausible that there is a mechanistic link between DXR, radiation therapy and our identified biomarkers. As previous research indicates, treatment of cHL contributes to an accelerated atherosclerotic process, resulting in the cardiovascular damage seen in our cHL cohort. In this setting, higher plasma levels of CYR61 could indicate the presence of ischemia, and ALCAM that the ischemia is secondary to atherosclerosis and vascular damage, where the lack of an adequate compensatory protective response as indicated by GPNMB, leads to manifest CVD over time. The lack of a statistical association with traditional biomarkers for manifest cardiovascular disease, such as CRP, Trop T and NT-proBNP, could be explained by the timing of the sampling in relation to treatment.

The present material is insufficient to perform a statistical analysis of individual outcomes or a dose response analysis, which could further strengthen our hypothesis. We suggest further studies in a larger cohort, preferably with the addition of standardized cardiac imaging, to show a dose‒response relationship between the identified biomarkers and the development of ischemic heart disease and HF. A larger study would also allow for the identification of potential cutoff levels associated with an increased risk of CVD. Prospective interventional studies, as proof of concept for biomarker-driven intervention, could lead to decreased cardiovascular morbidity in cHL survivors.

### Cardiovascular disease in cHL

In our cohort, we observed a significantly higher rate of risk factors for CVD and occurrence of CVD compared to our control cohort, despite the lower mean age among cHL patients, both at diagnosis and follow-up. However, all patients with CVD in the cHL cohort were older than 50 years of age. Furthermore, we did not observe the usual bimodal age distribution of cHL but a skewing toward younger patients in our cohort. Both of these effects should produce a trend toward a lower prevalence of CVD in the cHL cohort compared to controls. Closer contact with healthcare usually leads to a higher rate of uncovered subclinical disease, but the clear difference in the prevalence of the cHL cohort compared to the controls seems too large to be explained solely by such effects.

### Comparison with diffuse large B-cell lymphoma (DLBCL)

The potential protein biomarkers we identified for cHL did not show any significant correlation with CVD in our previous characterization of the plasma proteome in DLBCL patients [13]. Spondin-1 (SPON-1), which correlated with pretreatment CVD, and interleukin-1 receptor type 1 (IL-1RT1), which correlated with emerging CVD after treatment in DLBCL [13], did not show similar associations with the compound CVD endpoint in our cHL cohort. We believe these differences might be associated with the underlying mechanism of cardiac damage caused by the treatment of DLBCL and cHL. While the treatment regimens for DLBCL and cHL are both based on DXR, a considerable proportion of cHL patients also receive radiation therapy including the mediastinum and the cardiac damage caused by DXR in cHL is likely aggravated by radiation-induced damage [49].

### Study limitations

While PEA technology is a powerful tool for high-throughput screening for potential biomarkers in restricted sample volumes, the analysis is limited to the preselected available biomarkers in the panels. The PEA assay determines the relative abundance of proteins rather than absolute levels. Thus, we were not able to quantify the absolute levels of CYR61, GPNMB and ALCAM or propose suitable cutoffs due to insufficient remaining sample volumes in our biobank. The inconsistent biobank sample coverage in the cHL U-CAN cohort, alongside the limited sample size, reduces the predictive value. Nevertheless, the three identified biomarkers have possible unexplored potential based on statistical associations and plausible pathophysiological mechanisms. Our analysis may also fail to identify additional potential biomarkers present in the array due to the lack of statistical power. Building large cohorts is challenging due to the comparatively low prevalence of cHL. Our cohort represents the entire material that was available in the UCAN biobank at the time of analysis. The accumulated follow-up time of 7.8 years for our cohort should be compared to 15–20 years follow-up in historical cohorts, where mortality of late toxicity rivals the mortality of cHL [2, 3]. Therefore, while our analysis is well-suited to detect strong statistical correlations, weaker associations may go undetected due to the limited cohort size.

## Conclusion

We identified three possible soluble biomarkers, CYR61, GBNMB and ALCAM, associated with CVD in cHL. Higher levels of CYR61 indicate the presence of chronic ischemia, and ALCAM indicates increased atherosclerosis or vascular damage as the underlying cause leading to CVD in our cHL cohort. These findings are in line with previous evidence of the pathophysiological mechanisms of cardiotoxicity in cHL treatment. Higher levels of protective GPNMB indicate the activation of compensatory mechanisms to limit cardiac damage. The previous findings relating to SPON-1 and IL-1RT1 from DLCBL could not be reproduced.

While our analysis is limited by a small sample size and a comparatively short follow-up time, we believe that this exploratory analysis may have identified three possible biomarkers for the early prediction of cardiovascular morbidity in cHL patients. The association between CYR61, GPNMB and ALCAM will need to be verified independently in a prospective study or a separate biobank cohort. Should these biomarkers be confirmed to be associated with acute phase damage patients, they present an opportunity for early intervention before clinically manifest CVD has developed and may proceed biomarkers such as NT-proBNP and Trop T.

## Supplementary Information

Supplementary Material 1

## Data Availability

The datasets used and/or analyzed during the current study are available from the corresponding author on reasonable request.

## Abbreviations

ALCAM: Activated leukocyte cell adhesion molecule
BMI: Body mass index
BSA: Body surface area
cHL: Classical Hodgkin lymphoma
CVD: Cardiovascular disease
CRP: C-reactive protein
CYR61: Cysteine rich protein 61
DLBCL: Diffuse large B-cell lymphoma
DM: Diabetes mellitus
DNA: Deoxyribonucleic acid
DVT: Deep vein thrombosis
DXR: Doxorubicin
eGFR: Estimated glomerular filtration rate
GPNMB: Glycoprotein nonmetastatic melanoma protein B
HF: Heart failure
HT: Hypertension
HRQoL: Health-related quality of life
IPS: International Prognostic Score
LAD: Anterior descending artery
LOD: Limit of detection
NTpro-BNP: N-terminal pro-brain natriuretic peptide
NXP: Normalized Protein Expression
