# Supplementary material for: Exploratory study linking plasma proteomics to cardiotoxicity in Hodgkin lymphoma
Source: Cardiooncology. 2025 Dec 26;12:13. doi: 10.1186/s40959-025-00426-2 (PMC12853685; doi:10.1186/s40959-025-00426-2)
Supplement: Supplementary file 1 — Supplementary Material 1 [file 40959_2025_426_MOESM1_ESM.docx]

**Supplemental table 1:** Correlation between NXP values in samples collected before treatment, for all analyzed biomarkers in the CVD III and ONC II panels, and cardiovascular disease during follow up.

| **Protein** | **log2FC** | **SE** | **t.value** | **p** | **q** |
| --- | --- | --- | --- | --- | --- |
| CA9 | -0,79407 | 0,340836 | -2,32978 | 0,026083 | 0,990798 |
| FURIN | -0,29841 | 0,138401 | -2,15611 | 0,038461 | 0,990798 |
| MCP-1 | 0,516356 | 0,259604 | 1,989013 | 0,055307 | 0,990798 |
| ERBB4 | -0,26937 | 0,145595 | -1,85013 | 0,073266 | 0,990798 |
| vWF | 0,580109 | 0,322476 | 1,79892 | 0,081469 | 0,990798 |
| FAS | 0,395002 | 0,220435 | 1,791922 | 0,082607 | 0,990798 |
| Gal-4 | 0,483653 | 0,270156 | 1,790274 | 0,082876 | 0,990798 |
| CNTN1 | -0,42742 | 0,241913 | -1,76681 | 0,086797 | 0,990798 |
| CCL15 | 0,531354 | 0,304782 | 1,743389 | 0,090868 | 0,990798 |
| Ep-CAM | 0,94202 | 0,541219 | 1,740551 | 0,091372 | 0,990798 |
| CCL24 | -1,08717 | 0,648921 | -1,67534 | 0,103613 | 0,990798 |
| PGLYRP1 | -0,80937 | 0,506235 | -1,59879 | 0,119695 | 0,990798 |
| CXCL16 | 0,277907 | 0,17491 | 1,588853 | 0,121926 | 0,990798 |
| S100A11 | -0,37746 | 0,254564 | -1,48279 | 0,147619 | 0,990798 |
| MAD homolog 5 | -0,33311 | 0,224988 | -1,48055 | 0,14821 | 0,990798 |
| CPB1 | -0,4879 | 0,334441 | -1,45884 | 0,154355 | 0,990798 |
| TNFRSF10C | -0,57403 | 0,397907 | -1,44262 | 0,158844 | 0,990798 |
| Notch 3 | -0,3043 | 0,22251 | -1,36759 | 0,18097 | 0,990798 |
| CHIT1 | -1,8464 | 1,37135 | -1,34641 | 0,18763 | 0,990798 |
| LDL receptor | 0,609001 | 0,455972 | 1,335608 | 0,1911 | 0,990798 |
| uPA | -0,31855 | 0,238561 | -1,33531 | 0,191197 | 0,990798 |
| IGFBP-2 | 0,426351 | 0,326256 | 1,3068 | 0,200594 | 0,990798 |
| TCL1A | 0,940604 | 0,724719 | 1,297888 | 0,203325 | 0,990798 |
| VEGFR-3 | -0,15325 | 0,118744 | -1,29059 | 0,205815 | 0,990798 |
| ITGAV | -0,23537 | 0,186734 | -1,26048 | 0,216336 | 0,990798 |
| EGFR | -0,17574 | 0,141433 | -1,24255 | 0,223061 | 0,990798 |
| ADAM 8 | -0,38043 | 0,30888 | -1,23163 | 0,226793 | 0,990798 |
| ANXA1 | -0,44656 | 0,36381 | -1,22747 | 0,228331 | 0,990798 |
| PON3 | -0,75497 | 0,623145 | -1,21156 | 0,234554 | 0,990798 |
| PAI | -0,21365 | 0,176561 | -1,21007 | 0,235114 | 0,990798 |
| FR-alpha | 0,244637 | 0,202565 | 1,207695 | 0,235749 | 0,990798 |
| CTSZ | 0,322573 | 0,27519 | 1,172179 | 0,249778 | 0,990798 |
| SCF | -0,50801 | 0,443452 | -1,14559 | 0,260206 | 0,990798 |
| SPON1 | -0,19369 | 0,173373 | -1,11719 | 0,272231 | 0,990798 |
| CYR61 | 0,334271 | 0,300669 | 1,11176 | 0,274275 | 0,990798 |
| ITGB5 | -0,2083 | 0,187808 | -1,10914 | 0,27539 | 0,990798 |
| PDGF subunit A | -0,29527 | 0,268307 | -1,1005 | 0,279325 | 0,990798 |
| MB | -0,51518 | 0,468151 | -1,10047 | 0,279339 | 0,990798 |
| RET | -0,23008 | 0,213127 | -1,07954 | 0,288173 | 0,990798 |
| VIM | -0,41434 | 0,392298 | -1,05618 | 0,298555 | 0,990798 |
| FCRLB | -0,44653 | 0,43499 | -1,02653 | 0,312108 | 0,990798 |
| TR-AP | 0,331789 | 0,336115 | 0,987127 | 0,330986 | 0,990798 |
| SEZ6L | 0,172354 | 0,175902 | 0,979835 | 0,334297 | 0,990798 |
| SPARC | -0,10212 | 0,106655 | -0,95746 | 0,345298 | 0,990798 |
| DLK-1 | -0,36184 | 0,380776 | -0,95028 | 0,349093 | 0,990798 |
| CPE | -0,23219 | 0,245445 | -0,94598 | 0,351035 | 0,990798 |
| FGF-BP1 | -0,24041 | 0,256067 | -0,93887 | 0,35462 | 0,990798 |
| GPNMB | -0,09468 | 0,102192 | -0,92648 | 0,360922 | 0,990798 |
| CSTB | 0,499951 | 0,540336 | 0,925259 | 0,361757 | 0,990798 |
| FR-gamma | 1,028389 | 1,114462 | 0,922767 | 0,362827 | 0,990798 |
| GRN | -0,173 | 0,188196 | -0,91924 | 0,364848 | 0,990798 |
| IL-1RT2 | -0,31866 | 0,350115 | -0,91016 | 0,369541 | 0,990798 |
| IGFBP-7 | -0,24347 | 0,268515 | -0,90673 | 0,371327 | 0,990798 |
| HGF | -0,25985 | 0,29849 | -0,87054 | 0,390294 | 0,990798 |
| ICOSLG | -0,20204 | 0,232271 | -0,86983 | 0,390674 | 0,990798 |
| OPG | -0,21812 | 0,266807 | -0,81752 | 0,419678 | 0,990798 |
| RETN | -0,35883 | 0,444739 | -0,80683 | 0,425719 | 0,990798 |
| MK | -0,21086 | 0,261826 | -0,80533 | 0,426395 | 0,990798 |
| IL2-RA | -0,5977 | 0,751901 | -0,79492 | 0,432515 | 0,990798 |
| LYPD3 | -0,26571 | 0,337065 | -0,78832 | 0,436138 | 0,990798 |
| CCL16 | 0,245317 | 0,312367 | 0,785349 | 0,438026 | 0,990798 |
| SYND1 | 0,300134 | 0,392313 | 0,765039 | 0,449685 | 0,990798 |
| PCSK9 | -0,18286 | 0,243115 | -0,75214 | 0,457466 | 0,990798 |
| TGFR-2 | 0,16473 | 0,222573 | 0,740116 | 0,464463 | 0,990798 |
| AZU1 | -0,43487 | 0,598155 | -0,72703 | 0,472495 | 0,990798 |
| SELP | 0,201162 | 0,277781 | 0,724175 | 0,47422 | 0,990798 |
| CRNN | 0,370761 | 0,512263 | 0,723771 | 0,474306 | 0,990798 |
| TNF-R2 | 0,415028 | 0,579528 | 0,716149 | 0,479093 | 0,990798 |
| KLK6 | -0,15607 | 0,220746 | -0,70702 | 0,484672 | 0,990798 |
| XPNPEP2 | 0,296278 | 0,429265 | 0,690199 | 0,494895 | 0,990798 |
| AP-N | -0,13808 | 0,200299 | -0,68938 | 0,495556 | 0,990798 |
| MMP-9 | -0,27756 | 0,403189 | -0,68841 | 0,496156 | 0,990798 |
| RARRES2 | -0,10495 | 0,153687 | -0,68291 | 0,499581 | 0,990798 |
| COL1A1 | -0,14526 | 0,216086 | -0,67223 | 0,506263 | 0,990798 |
| CPA1 | -0,23667 | 0,358549 | -0,66008 | 0,513926 | 0,990798 |
| ERBB2 | -0,13345 | 0,202485 | -0,65904 | 0,514441 | 0,990798 |
| ST2 | 0,263247 | 0,405891 | 0,648565 | 0,521247 | 0,990798 |
| CEACAM5 | -0,25107 | 0,389713 | -0,64424 | 0,523875 | 0,990798 |
| DLL1 | 0,156223 | 0,24349 | 0,6416 | 0,525565 | 0,990798 |
| RSPO3 | -0,18241 | 0,293155 | -0,62222 | 0,538074 | 0,990798 |
| TNFRSF6B | 0,35348 | 0,589114 | 0,600019 | 0,552592 | 0,990798 |
| AR | 0,201064 | 0,337741 | 0,595318 | 0,555692 | 0,990798 |
| S100A4 | -0,11757 | 0,202407 | -0,58085 | 0,565285 | 0,990798 |
| MIA | 0,095757 | 0,167138 | 0,572925 | 0,570579 | 0,990798 |
| ESM-1 | 0,133706 | 0,236022 | 0,566498 | 0,574888 | 0,990798 |
| CD48 | -0,15654 | 0,278564 | -0,56197 | 0,577933 | 0,990798 |
| MMP-2 | -0,106 | 0,189668 | -0,55885 | 0,580156 | 0,990798 |
| FABP4 | 0,351738 | 0,630341 | 0,558013 | 0,580719 | 0,990798 |
| CEACAM1 | 0,043769 | 0,07901 | 0,553971 | 0,583333 | 0,990798 |
| TNFSF10 | -0,11045 | 0,203208 | -0,54354 | 0,590412 | 0,990798 |
| IL-6 | -0,55928 | 1,056082 | -0,52958 | 0,599949 | 0,990798 |
| PRTN3 | -0,278 | 0,531959 | -0,52259 | 0,604859 | 0,990798 |
| TIMP4 | 0,150971 | 0,291288 | 0,518286 | 0,607824 | 0,990798 |
| WIF-1 | 0,091228 | 0,179904 | 0,507094 | 0,615461 | 0,990798 |
| WISP-1 | -0,16667 | 0,329189 | -0,50632 | 0,615999 | 0,990798 |
| CD207 | -0,18654 | 0,369841 | -0,50437 | 0,61735 | 0,990798 |
| MMP-3 | -0,19273 | 0,384838 | -0,5008 | 0,61994 | 0,990798 |
| PPY | -0,41777 | 0,846224 | -0,49369 | 0,624797 | 0,990798 |
| PODXL | 0,057027 | 0,115642 | 0,493131 | 0,625186 | 0,990798 |
| TNFRSF19 | -0,12216 | 0,252809 | -0,48323 | 0,632124 | 0,990798 |
| LY9 | -0,14333 | 0,297217 | -0,48223 | 0,632826 | 0,990798 |
| TLT-2 | -0,13823 | 0,291282 | -0,47457 | 0,638316 | 0,990798 |
| MSLN | 0,162403 | 0,343576 | 0,472685 | 0,639549 | 0,990798 |
| hK8 | -0,11333 | 0,240765 | -0,4707 | 0,640953 | 0,990798 |
| ADAM-TS 15 | 0,11323 | 0,244541 | 0,463031 | 0,646381 | 0,990798 |
| hK14 | 0,158247 | 0,34688 | 0,456202 | 0,651233 | 0,990798 |
| FASLG | -0,13181 | 0,29239 | -0,45081 | 0,655078 | 0,990798 |
| MEPE | -0,11728 | 0,266879 | -0,43945 | 0,66329 | 0,990798 |
| EPHA2 | 0,106042 | 0,243164 | 0,436092 | 0,665609 | 0,990798 |
| U-PAR | -0,16709 | 0,387016 | -0,43174 | 0,668824 | 0,990798 |
| TLR3 | 0,183548 | 0,427657 | 0,429195 | 0,67057 | 0,990798 |
| TGF-alpha | -0,26025 | 0,609215 | -0,42719 | 0,672012 | 0,990798 |
| EPHB4 | 0,146692 | 0,344315 | 0,426041 | 0,67293 | 0,990798 |
| MetAP 2 | 0,119385 | 0,300053 | 0,397879 | 0,693282 | 0,990798 |
| PECAM-1 | 0,099448 | 0,250372 | 0,3972 | 0,693857 | 0,990798 |
| CD93 | 0,068556 | 0,179786 | 0,381322 | 0,705485 | 0,990798 |
| CD70 | -0,18249 | 0,488041 | -0,37391 | 0,71086 | 0,990798 |
| 5'-NT | 0,096555 | 0,268805 | 0,359202 | 0,721733 | 0,990798 |
| AXL | -0,11091 | 0,309263 | -0,35863 | 0,72223 | 0,990798 |
| TXLNA | -0,15818 | 0,44384 | -0,35638 | 0,723823 | 0,990798 |
| EGF | 0,185848 | 0,543427 | 0,341992 | 0,734525 | 0,990798 |
| KLK13 | 0,118283 | 0,352009 | 0,336021 | 0,738982 | 0,990798 |
| CD163 | -0,12169 | 0,368579 | -0,33016 | 0,743429 | 0,990798 |
| MUC-16 | -0,20322 | 0,62047 | -0,32753 | 0,745336 | 0,990798 |
| ALCAM | -0,06868 | 0,215676 | -0,31845 | 0,752211 | 0,990798 |
| WFDC2 | -0,06984 | 0,219992 | -0,31748 | 0,75288 | 0,990798 |
| TR | -0,13006 | 0,443415 | -0,29332 | 0,771174 | 0,990798 |
| CTSD | 0,08274 | 0,286879 | 0,288414 | 0,77489 | 0,990798 |
| hK11 | -0,05736 | 0,200583 | -0,28597 | 0,77669 | 0,990798 |
| CD160 | -0,10456 | 0,366695 | -0,28514 | 0,777319 | 0,990798 |
| MIC-A/B | -0,21427 | 0,751617 | -0,28508 | 0,777363 | 0,990798 |
| GZMB | 0,156681 | 0,553215 | 0,283218 | 0,778779 | 0,990798 |
| SCAMP3 | 0,15072 | 0,539199 | 0,279526 | 0,781586 | 0,990798 |
| GZMH | -0,15901 | 0,634544 | -0,25058 | 0,803691 | 0,990798 |
| SELE | -0,10685 | 0,44751 | -0,23876 | 0,812817 | 0,990798 |
| IGFBP-1 | 0,212147 | 0,91823 | 0,23104 | 0,818755 | 0,990798 |
| SCGB3A2 | 0,103921 | 0,453027 | 0,229392 | 0,820024 | 0,990798 |
| Gal-3 | 0,06429 | 0,280904 | 0,228868 | 0,820428 | 0,990798 |
| CTSV | -0,09452 | 0,430107 | -0,21976 | 0,827415 | 0,990798 |
| t-PA | 0,09881 | 0,451693 | 0,218754 | 0,828231 | 0,990798 |
| MPO | 0,074961 | 0,354869 | 0,211236 | 0,834043 | 0,990798 |
| FADD | -0,08384 | 0,403929 | -0,20757 | 0,836842 | 0,990798 |
| TNFSF13B | -0,08004 | 0,38825 | -0,20615 | 0,837979 | 0,990798 |
| GPC1 | 0,045322 | 0,224812 | 0,201601 | 0,841466 | 0,990798 |
| ERBB3 | -0,02688 | 0,134629 | -0,19963 | 0,842998 | 0,990798 |
| IFN-gamma-R1 | 0,04958 | 0,252784 | 0,196135 | 0,845708 | 0,990798 |
| IGF1R | -0,04741 | 0,251924 | -0,18818 | 0,851887 | 0,990798 |
| TNF-R1 | 0,078301 | 0,445857 | 0,175619 | 0,8617 | 0,990798 |
| PLC | -0,03236 | 0,203994 | -0,15862 | 0,874966 | 0,990798 |
| ITGB2 | -0,04829 | 0,305626 | -0,15799 | 0,875455 | 0,990798 |
| PI3 | -0,06329 | 0,421958 | -0,15 | 0,881706 | 0,990798 |
| PVRL4 | -0,04465 | 0,302834 | -0,14745 | 0,883674 | 0,990798 |
| CHI3L1 | 0,090479 | 0,62181 | 0,14551 | 0,885221 | 0,990798 |
| Gal-1 | 0,024587 | 0,170099 | 0,144545 | 0,885949 | 0,990798 |
| LYN | -0,02536 | 0,176193 | -0,14393 | 0,886433 | 0,990798 |
| ICAM-2 | 0,038778 | 0,291403 | 0,133074 | 0,894968 | 0,990798 |
| VEGFR-2 | -0,01669 | 0,126099 | -0,13235 | 0,895508 | 0,990798 |
| CRPvalue | -0,13875 | 1,144064 | -0,12128 | 0,90399 | 0,990798 |
| IL-17RA | -0,03266 | 0,268856 | -0,12147 | 0,904076 | 0,990798 |
| LTBR | -0,0369 | 0,317552 | -0,11619 | 0,908226 | 0,990798 |
| CD27 | 0,034995 | 0,301757 | 0,115972 | 0,908378 | 0,990798 |
| IL-1RT1 | -0,01682 | 0,169555 | -0,09921 | 0,921587 | 0,990798 |
| TNFRSF4 | 0,069522 | 0,716433 | 0,097039 | 0,923282 | 0,990798 |
| CXL17 | -0,02808 | 0,292422 | -0,09602 | 0,924085 | 0,990798 |
| GDF-15 | -0,0529 | 0,629454 | -0,08404 | 0,933547 | 0,990798 |
| IL-6RA | 0,017689 | 0,21598 | 0,081901 | 0,935235 | 0,990798 |
| ABL1 | -0,02278 | 0,359762 | -0,06331 | 0,949902 | 0,990798 |
| SHPS-1 | -0,02565 | 0,425535 | -0,06027 | 0,952318 | 0,990798 |
| JAM-A | -0,01539 | 0,257861 | -0,05969 | 0,952771 | 0,990798 |
| TFPI-2 | 0,030223 | 0,51029 | 0,059228 | 0,953128 | 0,990798 |
| CASP-3 | -0,02377 | 0,406216 | -0,05852 | 0,953701 | 0,990798 |
| TNFRSF14 | -0,02588 | 0,469288 | -0,05514 | 0,956372 | 0,990798 |
| TNFSF13 | -0,01134 | 0,205962 | -0,05508 | 0,956409 | 0,990798 |
| OPN | 0,01617 | 0,336463 | 0,048058 | 0,961969 | 0,990798 |
| PSP-D | 0,020827 | 0,452271 | 0,046049 | 0,963557 | 0,990798 |
| TFF3 | -0,02287 | 0,559505 | -0,04087 | 0,967653 | 0,990798 |
| DKN1A | -0,01791 | 0,46627 | -0,03841 | 0,969589 | 0,990798 |
| VEGF-A | 0,009646 | 0,355224 | 0,027156 | 0,978499 | 0,990798 |
| IL-18BP | -0,00959 | 0,418673 | -0,02289 | 0,981877 | 0,990798 |
| CXCL13 | 0,008284 | 0,428577 | 0,01933 | 0,984694 | 0,990798 |
| CDH5 | -0,00473 | 0,248297 | -0,01906 | 0,984909 | 0,990798 |
| TFPI | 0,002853 | 0,154493 | 0,018464 | 0,985384 | 0,990798 |
| BLM hydrolase | -0,00399 | 0,389432 | -0,01025 | 0,991888 | 0,991888 |
| **Protein** | **log2FC** | **SE** | **t.value** | **p** | **q** |
| CPB1 | 0,775336 | 0,250188 | 3,09901 | 0,004028 | 0,653495 |
| CPA1 | 0,763992 | 0,265813 | 2,874174 | 0,007142 | 0,653495 |
| TNFRSF10C | -0,80483 | 0,307889 | -2,61401 | 0,013528 | 0,825209 |
| PI3 | 0,779411 | 0,32026 | 2,43368 | 0,020709 | 0,947449 |
| MMP-9 | -0,66899 | 0,313936 | -2,13099 | 0,040871 | 0,982345 |
| IL-17RA | -0,43658 | 0,208275 | -2,09616 | 0,044064 | 0,982345 |
| ADAM 8 | -0,50736 | 0,245035 | -2,07055 | 0,046299 | 0,982345 |
| ANXA1 | -0,59666 | 0,28862 | -2,06729 | 0,046624 | 0,982345 |
| EGFR | -0,22317 | 0,112909 | -1,97655 | 0,056765 | 0,982345 |
| TGF-alpha | -0,92753 | 0,477116 | -1,94403 | 0,060458 | 0,982345 |
| MB | -0,71013 | 0,373383 | -1,90188 | 0,066221 | 0,982345 |
| WISP-1 | -0,48162 | 0,259256 | -1,85771 | 0,072153 | 0,982345 |
| AR | 0,469452 | 0,267773 | 1,753172 | 0,088859 | 0,982345 |
| S100A4 | -0,27321 | 0,160856 | -1,69845 | 0,098834 | 0,982345 |
| ITGB2 | -0,41066 | 0,241873 | -1,69783 | 0,099246 | 0,982345 |
| AZU1 | -0,80153 | 0,477535 | -1,67847 | 0,102997 | 0,982345 |
| U-PAR | -0,49744 | 0,308291 | -1,61355 | 0,116445 | 0,982345 |
| CEACAM5 | 0,490411 | 0,311867 | 1,572502 | 0,125374 | 0,982345 |
| CHIT1 | -1,67077 | 1,126232 | -1,4835 | 0,147725 | 0,982345 |
| TCL1A | -0,87054 | 0,593567 | -1,46663 | 0,151944 | 0,982345 |
| CA9 | 0,423558 | 0,294158 | 1,4399 | 0,159316 | 0,982345 |
| S100A11 | -0,30038 | 0,210372 | -1,42784 | 0,162733 | 0,982345 |
| Gal-1 | -0,19177 | 0,136265 | -1,40729 | 0,16869 | 0,982345 |
| GPC1 | -0,24946 | 0,18032 | -1,38341 | 0,175828 | 0,982345 |
| CXCL16 | -0,1976 | 0,145936 | -1,35403 | 0,185214 | 0,982345 |
| LYPD3 | -0,36138 | 0,273377 | -1,32192 | 0,195284 | 0,982345 |
| CSTB | -0,58204 | 0,440363 | -1,32172 | 0,195632 | 0,982345 |
| MSLN | 0,361152 | 0,277203 | 1,302842 | 0,201647 | 0,982345 |
| PDGF subunit A | -0,28652 | 0,22001 | -1,30229 | 0,202112 | 0,982345 |
| HGF | -0,31127 | 0,242952 | -1,28119 | 0,209059 | 0,982345 |
| FR-gamma | -1,16221 | 0,908419 | -1,27938 | 0,209686 | 0,982345 |
| CXL17 | 0,279675 | 0,236176 | 1,18418 | 0,244801 | 0,982345 |
| IGF1R | -0,23862 | 0,203634 | -1,17181 | 0,249663 | 0,982345 |
| ABL1 | -0,3348 | 0,290867 | -1,15104 | 0,257987 | 0,982345 |
| CD160 | 0,341214 | 0,296835 | 1,149506 | 0,25861 | 0,982345 |
| ITGAV | -0,17742 | 0,154573 | -1,14782 | 0,259297 | 0,982345 |
| SELE | -0,41137 | 0,362735 | -1,13409 | 0,26518 | 0,982345 |
| PRTN3 | -0,48731 | 0,432755 | -1,12607 | 0,26851 | 0,982345 |
| IGFBP-7 | -0,24752 | 0,220309 | -1,12351 | 0,269581 | 0,982345 |
| FCRLB | -0,39141 | 0,357911 | -1,0936 | 0,282048 | 0,982345 |
| ICOSLG | 0,207684 | 0,190291 | 1,091406 | 0,282999 | 0,982345 |
| PLC | -0,18049 | 0,165508 | -1,09055 | 0,283616 | 0,982345 |
| TLR3 | 0,374559 | 0,34753 | 1,077775 | 0,28895 | 0,982345 |
| SCGB3A2 | -0,39408 | 0,367951 | -1,071 | 0,292181 | 0,982345 |
| uPA | -0,21225 | 0,198949 | -1,06684 | 0,294025 | 0,982345 |
| PGLYRP1 | -0,44863 | 0,427208 | -1,05014 | 0,301521 | 0,982345 |
| SELP | -0,23824 | 0,227441 | -1,04748 | 0,302728 | 0,982345 |
| KLK6 | 0,187955 | 0,180716 | 1,040061 | 0,306106 | 0,982345 |
| VIM | -0,3342 | 0,323692 | -1,03245 | 0,309368 | 0,982345 |
| TLT-2 | -0,24226 | 0,237602 | -1,01962 | 0,315554 | 0,982345 |
| MCP-1 | -0,22317 | 0,22384 | -0,997 | 0,326245 | 0,982345 |
| CEACAM1 | -0,06225 | 0,064544 | -0,96448 | 0,341821 | 0,982345 |
| XPNPEP2 | -0,33581 | 0,351657 | -0,95495 | 0,346546 | 0,982345 |
| TNF-R1 | -0,34419 | 0,363377 | -0,94719 | 0,350639 | 0,982345 |
| CD93 | -0,13659 | 0,146861 | -0,93009 | 0,359288 | 0,982345 |
| IGFBP-1 | -0,69277 | 0,749105 | -0,9248 | 0,361992 | 0,982345 |
| GRN | -0,14278 | 0,155444 | -0,91852 | 0,365217 | 0,982345 |
| MAD homolog 5 | 0,170152 | 0,189266 | 0,899013 | 0,375159 | 0,982345 |
| FADD | -0,29499 | 0,329285 | -0,89585 | 0,376823 | 0,982345 |
| MetAP 2 | -0,2098 | 0,245288 | -0,85531 | 0,398547 | 0,982345 |
| COL1A1 | -0,15199 | 0,177714 | -0,85524 | 0,398775 | 0,982345 |
| IL-1RT2 | -0,24654 | 0,289638 | -0,8512 | 0,400982 | 0,982345 |
| MPO | -0,24349 | 0,290135 | -0,83922 | 0,407573 | 0,982345 |
| CDH5 | -0,16965 | 0,202879 | -0,83619 | 0,40925 | 0,982345 |
| MEPE | -0,18022 | 0,218787 | -0,82373 | 0,416192 | 0,982345 |
| MIC-A/B | -0,49824 | 0,614393 | -0,81095 | 0,423207 | 0,982345 |
| LYN | -0,11465 | 0,143943 | -0,79652 | 0,431422 | 0,982345 |
| CXCL13 | 0,278174 | 0,350039 | 0,794697 | 0,432469 | 0,982345 |
| hK11 | -0,1298 | 0,164041 | -0,79129 | 0,434428 | 0,982345 |
| TNFRSF6B | -0,38205 | 0,483829 | -0,78964 | 0,435378 | 0,982345 |
| IL-6 | 0,682724 | 0,866338 | 0,788057 | 0,436288 | 0,982345 |
| MK | -0,16801 | 0,216021 | -0,77775 | 0,442259 | 0,982345 |
| SCAMP3 | -0,34149 | 0,441118 | -0,77414 | 0,444361 | 0,982345 |
| MIA | 0,105724 | 0,137264 | 0,770224 | 0,446646 | 0,982345 |
| TIMP4 | 0,184095 | 0,239397 | 0,768994 | 0,447536 | 0,982345 |
| WFDC2 | 0,133057 | 0,180182 | 0,738457 | 0,465457 | 0,982345 |
| TNFRSF4 | -0,42164 | 0,58622 | -0,71926 | 0,477046 | 0,982345 |
| CASP-3 | -0,23791 | 0,332888 | -0,71468 | 0,479988 | 0,982345 |
| IL-6RA | -0,12489 | 0,177037 | -0,70545 | 0,485637 | 0,982345 |
| TNFRSF14 | -0,27073 | 0,384664 | -0,70381 | 0,486638 | 0,982345 |
| TNFRSF19 | -0,14534 | 0,207647 | -0,69992 | 0,488881 | 0,982345 |
| IL2-RA | -0,43141 | 0,622485 | -0,69304 | 0,493282 | 0,982345 |
| CCL16 | 0,176785 | 0,258594 | 0,683638 | 0,499125 | 0,982345 |
| CRNN | 0,285303 | 0,422803 | 0,674789 | 0,50451 | 0,982345 |
| MMP-3 | 0,211195 | 0,31691 | 0,666419 | 0,50992 | 0,982345 |
| BLM hydrolase | -0,21164 | 0,319471 | -0,66249 | 0,512403 | 0,982345 |
| TR | -0,23599 | 0,364353 | -0,6477 | 0,521799 | 0,982345 |
| CTSV | -0,22724 | 0,352679 | -0,64434 | 0,523812 | 0,982345 |
| FAS | -0,12227 | 0,189759 | -0,64436 | 0,523935 | 0,982345 |
| SCF | -0,23788 | 0,370527 | -0,642 | 0,525311 | 0,982345 |
| CCL15 | -0,16693 | 0,261763 | -0,63771 | 0,528198 | 0,982345 |
| GZMH | -0,33128 | 0,520506 | -0,63647 | 0,528863 | 0,982345 |
| DKN1A | -0,23882 | 0,382203 | -0,62486 | 0,536362 | 0,982345 |
| SPARC | -0,05494 | 0,088637 | -0,61979 | 0,539652 | 0,982345 |
| CTSZ | -0,14188 | 0,230765 | -0,61485 | 0,543003 | 0,982345 |
| EPHA2 | -0,12135 | 0,199957 | -0,60689 | 0,548079 | 0,982345 |
| RSPO3 | -0,14638 | 0,241787 | -0,6054 | 0,549053 | 0,982345 |
| TXLNA | -0,21556 | 0,364733 | -0,59102 | 0,558536 | 0,982345 |
| TNFSF13 | 0,096916 | 0,168987 | 0,573512 | 0,570186 | 0,982345 |
| hK8 | 0,113614 | 0,198196 | 0,57324 | 0,570368 | 0,982345 |
| DLK-1 | 0,179138 | 0,317334 | 0,564509 | 0,576344 | 0,982345 |
| AXL | 0,142151 | 0,254714 | 0,55808 | 0,580674 | 0,982345 |
| SEZ6L | 0,081427 | 0,146444 | 0,556028 | 0,581942 | 0,982345 |
| GZMB | -0,2518 | 0,454582 | -0,55391 | 0,583376 | 0,982345 |
| MMP-2 | -0,08433 | 0,156712 | -0,53814 | 0,594202 | 0,982345 |
| JAM-A | -0,11317 | 0,212052 | -0,53369 | 0,597246 | 0,982345 |
| CD27 | -0,13052 | 0,247815 | -0,52669 | 0,601928 | 0,982345 |
| t-PA | 0,187789 | 0,371879 | 0,504974 | 0,617037 | 0,982345 |
| TNF-R2 | -0,24245 | 0,480578 | -0,50449 | 0,61737 | 0,982345 |
| PPY | 0,350589 | 0,697636 | 0,502538 | 0,618626 | 0,982345 |
| RET | -0,08816 | 0,178143 | -0,49486 | 0,623977 | 0,982345 |
| FABP4 | -0,25637 | 0,521194 | -0,49189 | 0,626152 | 0,982345 |
| RETN | -0,18168 | 0,36966 | -0,49149 | 0,626434 | 0,982345 |
| GPNMB | 0,041675 | 0,085039 | 0,490074 | 0,627324 | 0,982345 |
| VEGFR-3 | 0,048023 | 0,099998 | 0,480243 | 0,634223 | 0,982345 |
| LTBR | -0,12471 | 0,261411 | -0,47705 | 0,636567 | 0,982345 |
| PSP-D | 0,174649 | 0,37229 | 0,469121 | 0,642164 | 0,982345 |
| RARRES2 | -0,05907 | 0,127433 | -0,46351 | 0,646136 | 0,982345 |
| TGFR-2 | -0,08507 | 0,184439 | -0,46126 | 0,647637 | 0,982345 |
| Ep-CAM | -0,20546 | 0,466293 | -0,44063 | 0,662442 | 0,982345 |
| TNFSF10 | -0,07089 | 0,167844 | -0,42236 | 0,675499 | 0,982345 |
| PON3 | -0,22118 | 0,524908 | -0,42137 | 0,6763 | 0,982345 |
| TNFSF13B | 0,133908 | 0,320016 | 0,41844 | 0,67842 | 0,982345 |
| CD70 | 0,16565 | 0,402219 | 0,41184 | 0,683119 | 0,982345 |
| EPHB4 | -0,11586 | 0,284458 | -0,40731 | 0,68649 | 0,982345 |
| CD207 | 0,122909 | 0,305366 | 0,402498 | 0,689913 | 0,982345 |
| VEGF-A | 0,117362 | 0,292179 | 0,401678 | 0,69051 | 0,982345 |
| TFF3 | -0,17291 | 0,461127 | -0,37497 | 0,710154 | 0,982345 |
| AP-N | -0,06178 | 0,166303 | -0,3715 | 0,712717 | 0,982345 |
| WIF-1 | -0,0535 | 0,14862 | -0,35996 | 0,721168 | 0,982345 |
| TFPI | -0,04405 | 0,127367 | -0,34589 | 0,73169 | 0,982345 |
| ESM-1 | -0,06729 | 0,195197 | -0,34473 | 0,732483 | 0,982345 |
| PCSK9 | 0,068671 | 0,202205 | 0,339609 | 0,73637 | 0,982345 |
| ERBB2 | -0,0568 | 0,167757 | -0,33857 | 0,737082 | 0,982345 |
| CYR61 | -0,08265 | 0,252097 | -0,32786 | 0,745089 | 0,982345 |
| PAI | -0,04787 | 0,14889 | -0,32151 | 0,749917 | 0,982345 |
| ALCAM | -0,05662 | 0,17814 | -0,31783 | 0,752681 | 0,982345 |
| MUC-16 | -0,15064 | 0,511749 | -0,29436 | 0,770329 | 0,982345 |
| SHPS-1 | -0,10218 | 0,351029 | -0,29108 | 0,772868 | 0,982345 |
| TFPI-2 | 0,119678 | 0,42025 | 0,284779 | 0,777593 | 0,982345 |
| ERBB3 | -0,03065 | 0,110943 | -0,27627 | 0,784061 | 0,982345 |
| ST2 | -0,09312 | 0,337042 | -0,27629 | 0,784102 | 0,982345 |
| LDL receptor | 0,106118 | 0,386513 | 0,274552 | 0,785425 | 0,982345 |
| CD48 | 0,062623 | 0,23052 | 0,271658 | 0,787578 | 0,982345 |
| FASLG | -0,06538 | 0,241554 | -0,27066 | 0,788338 | 0,982345 |
| vWF | -0,07549 | 0,279176 | -0,27042 | 0,788575 | 0,982345 |
| GDF-15 | 0,135864 | 0,519404 | 0,261577 | 0,795324 | 0,982345 |
| OPG | -0,05501 | 0,222448 | -0,24731 | 0,806245 | 0,982345 |
| EGF | 0,103455 | 0,448498 | 0,230669 | 0,818996 | 0,982345 |
| CPE | 0,047096 | 0,204936 | 0,229811 | 0,819657 | 0,982345 |
| FR-alpha | 0,035304 | 0,170559 | 0,206992 | 0,837288 | 0,982345 |
| CCL24 | -0,11106 | 0,558647 | -0,1988 | 0,84368 | 0,982345 |
| KLK13 | 0,054658 | 0,290578 | 0,1881 | 0,85195 | 0,982345 |
| ITGB5 | -0,02956 | 0,157627 | -0,18753 | 0,852393 | 0,982345 |
| CD163 | 0,056067 | 0,304787 | 0,183956 | 0,855208 | 0,982345 |
| PECAM-1 | -0,03807 | 0,207196 | -0,18376 | 0,855362 | 0,982345 |
| IGFBP-2 | 0,050573 | 0,276425 | 0,182952 | 0,85599 | 0,982345 |
| TR-AP | 0,049329 | 0,281677 | 0,175126 | 0,862084 | 0,982345 |
| PODXL | -0,01666 | 0,095656 | -0,17417 | 0,862796 | 0,982345 |
| VEGFR-2 | -0,01753 | 0,103954 | -0,16862 | 0,867128 | 0,982345 |
| PVRL4 | -0,04034 | 0,249676 | -0,16158 | 0,872622 | 0,982345 |
| ERBB4 | -0,02026 | 0,126069 | -0,16074 | 0,873278 | 0,982345 |
| FURIN | 0,019164 | 0,121841 | 0,157283 | 0,87598 | 0,982345 |
| FGF-BP1 | 0,030282 | 0,213868 | 0,141591 | 0,888264 | 0,982345 |
| Gal-4 | 0,032244 | 0,233975 | 0,137809 | 0,891255 | 0,982345 |
| 5'-NT | 0,029411 | 0,222009 | 0,132474 | 0,895413 | 0,982345 |
| hK14 | 0,036864 | 0,286838 | 0,128517 | 0,89852 | 0,982345 |
| Gal-3 | -0,02464 | 0,232163 | -0,10614 | 0,916135 | 0,982345 |
| CTSD | 0,023889 | 0,23722 | 0,100705 | 0,920413 | 0,982345 |
| CRPvalue | 0,09559 | 0,964723 | 0,099085 | 0,921492 | 0,982345 |
| LY9 | -0,02367 | 0,245889 | -0,09626 | 0,923895 | 0,982345 |
| IFN-gamma-R1 | -0,01962 | 0,208518 | -0,09409 | 0,925609 | 0,982345 |
| Notch 3 | 0,017059 | 0,189054 | 0,090233 | 0,928665 | 0,982345 |
| ADAM-TS 15 | 0,015061 | 0,202266 | 0,074459 | 0,941095 | 0,984136 |
| IL-18BP | 0,023572 | 0,345783 | 0,068171 | 0,946074 | 0,984136 |
| IL-1RT1 | -0,00947 | 0,140057 | -0,06764 | 0,946492 | 0,984136 |
| CNTN1 | -0,01181 | 0,209318 | -0,05644 | 0,955341 | 0,987726 |
| CHI3L1 | 0,012352 | 0,513753 | 0,024042 | 0,980968 | 0,997753 |
| SYND1 | 0,005889 | 0,326324 | 0,018047 | 0,98571 | 0,997753 |
| ICAM-2 | 0,003362 | 0,240752 | 0,013963 | 0,988946 | 0,997753 |
| OPN | -0,00377 | 0,277913 | -0,01356 | 0,989265 | 0,997753 |
| SPON1 | 0,00137 | 0,145964 | 0,009388 | 0,992568 | 0,997753 |
| DLL1 | 0,000573 | 0,20201 | 0,002838 | 0,997753 | 0,997753 |

**Supplemental table 2:** Correlation between NXP values in samples collected at follow up after treatment, for all analyzed biomarkers in the CVD III and ONC II panels, and cardiovascular disease during follow up.

| **Protein** | **log2FC** | **SE** | **t.value** | **p** | **q** |
| --- | --- | --- | --- | --- | --- |
| CYR61 | 1,126805 | 0,199726 | 5,641765 | 4,69E-05 | 0,008624 |
| GPNMB | 0,28544 | 0,068046 | 4,194803 | 0,000781 | 0,054063 |
| ALCAM | 0,480909 | 0,116302 | 4,135016 | 0,000881 | 0,054063 |
| ADAM 8 | 0,329935 | 0,096405 | 3,422381 | 0,00378 | 0,154053 |
| AXL | 0,693093 | 0,20748 | 3,340534 | 0,004471 | 0,154053 |
| TNFSF13B | 1,200265 | 0,365516 | 3,283753 | 0,005023 | 0,154053 |
| TNFSF13 | 0,502074 | 0,157943 | 3,178839 | 0,006228 | 0,163703 |
| PSP-D | 0,953265 | 0,321011 | 2,969569 | 0,009546 | 0,173669 |
| Gal-1 | 0,205401 | 0,069254 | 2,965931 | 0,009617 | 0,173669 |
| CEACAM5 | 1,009883 | 0,344756 | 2,929266 | 0,010361 | 0,173669 |
| FAS | 0,362698 | 0,123862 | 2,928239 | 0,010382 | 0,173669 |
| Notch 3 | 0,622769 | 0,220281 | 2,827155 | 0,012742 | 0,19015 |
| ADAM-TS 15 | 0,642813 | 0,2295 | 2,800928 | 0,013435 | 0,19015 |
| ERBB2 | 0,586779 | 0,216746 | 2,707216 | 0,016222 | 0,193594 |
| CNTN1 | 0,320786 | 0,12076 | 2,656383 | 0,017961 | 0,193594 |
| ICOSLG | 0,305613 | 0,116077 | 2,632838 | 0,018826 | 0,193594 |
| GDF-15 | 0,640398 | 0,249748 | 2,564175 | 0,021582 | 0,193594 |
| LY9 | 0,567045 | 0,221175 | 2,56378 | 0,021599 | 0,193594 |
| CXL17 | 0,616995 | 0,240845 | 2,561793 | 0,021685 | 0,193594 |
| KLK6 | 0,461895 | 0,180306 | 2,561731 | 0,021687 | 0,193594 |
| IFN-gamma-R1 | 0,337504 | 0,132233 | 2,552345 | 0,022095 | 0,193594 |
| TR-AP | 0,416906 | 0,1649 | 2,528236 | 0,023176 | 0,193834 |
| MAD homolog 5 | 0,349326 | 0,140189 | 2,491815 | 0,024905 | 0,199238 |
| PODXL | 0,202117 | 0,083149 | 2,430774 | 0,028081 | 0,215289 |
| TLR3 | 0,639419 | 0,266288 | 2,401234 | 0,029753 | 0,218983 |
| FURIN | 0,384978 | 0,161927 | 2,377485 | 0,031165 | 0,219782 |
| DLK-1 | 1,013258 | 0,440449 | 2,300511 | 0,036189 | 0,219782 |
| MMP-2 | 0,363773 | 0,158287 | 2,29819 | 0,036351 | 0,219782 |
| VEGFR-2 | 0,252485 | 0,110237 | 2,290393 | 0,036903 | 0,219782 |
| MCP-1 | 0,414949 | 0,181698 | 2,283729 | 0,03738 | 0,219782 |
| VEGFR-3 | 0,179236 | 0,078501 | 2,283237 | 0,037416 | 0,219782 |
| SCF | 0,765932 | 0,337093 | 2,272167 | 0,038223 | 0,219782 |
| SPARC | 0,210308 | 0,09729 | 2,161652 | 0,04722 | 0,261937 |
| RET | 0,512333 | 0,238447 | 2,148619 | 0,048401 | 0,261937 |
| TNFRSF19 | 0,403715 | 0,190397 | 2,120388 | 0,051056 | 0,268407 |
| SEZ6L | 0,309052 | 0,148452 | 2,081835 | 0,054897 | 0,280586 |
| U-PAR | 0,460104 | 0,225222 | 2,042891 | 0,059046 | 0,286193 |
| TCL1A | 0,722464 | 0,358146 | 2,017232 | 0,061934 | 0,286193 |
| OPN | 0,625461 | 0,311605 | 2,007221 | 0,063095 | 0,286193 |
| ERBB4 | 0,299971 | 0,14962 | 2,004884 | 0,063369 | 0,286193 |
| ITGB5 | 0,360848 | 0,180291 | 2,00147 | 0,063771 | 0,286193 |
| CD93 | 0,384191 | 0,195273 | 1,967455 | 0,067906 | 0,293016 |
| WFDC2 | 0,416317 | 0,212552 | 1,958661 | 0,069014 | 0,293016 |
| hK14 | 0,445392 | 0,228359 | 1,950404 | 0,070069 | 0,293016 |
| CD160 | 0,724578 | 0,376208 | 1,926003 | 0,073273 | 0,294626 |
| MB | 0,894677 | 0,465216 | 1,923143 | 0,073656 | 0,294626 |
| TNFSF10 | 0,329902 | 0,17696 | 1,864272 | 0,081973 | 0,304931 |
| KLK13 | 0,581421 | 0,316727 | 1,835718 | 0,086302 | 0,304931 |
| MSLN | 0,801091 | 0,437848 | 1,82961 | 0,087254 | 0,304931 |
| TFPI-2 | 0,378516 | 0,206957 | 1,828955 | 0,087357 | 0,304931 |
| CCL16 | 0,440525 | 0,241444 | 1,82454 | 0,088051 | 0,304931 |
| 5'-NT | 0,431977 | 0,237934 | 1,815536 | 0,089483 | 0,304931 |
| ST2 | 0,556579 | 0,307409 | 1,810552 | 0,090284 | 0,304931 |
| EGFR | 0,240946 | 0,135126 | 1,783128 | 0,094809 | 0,304931 |
| CEACAM1 | 0,109528 | 0,061903 | 1,769349 | 0,097157 | 0,304931 |
| ITGAV | 0,301924 | 0,171434 | 1,761164 | 0,098576 | 0,304931 |
| SHPS-1 | 0,395713 | 0,224798 | 1,760308 | 0,098725 | 0,304931 |
| AP-N | 0,221825 | 0,126481 | 1,753813 | 0,099865 | 0,304931 |
| MIA | 0,13787 | 0,078633 | 1,75334 | 0,099949 | 0,304931 |
| RARRES2 | 0,155899 | 0,089192 | 1,7479 | 0,100914 | 0,304931 |
| COL1A1 | 0,409303 | 0,234302 | 1,746904 | 0,101091 | 0,304931 |
| CPE | 0,358141 | 0,210134 | 1,704342 | 0,108937 | 0,3125 |
| TNFRSF10C | 0,386037 | 0,227169 | 1,699337 | 0,109893 | 0,3125 |
| IL-6 | 0,842478 | 0,496493 | 1,696859 | 0,11037 | 0,3125 |
| WIF-1 | 0,319613 | 0,189576 | 1,685934 | 0,112491 | 0,3125 |
| LDL receptor | 0,477171 | 0,283829 | 1,681195 | 0,113423 | 0,3125 |
| TGFR-2 | 0,285966 | 0,170286 | 1,67933 | 0,113791 | 0,3125 |
| LYPD3 | 0,526076 | 0,316179 | 1,663854 | 0,116887 | 0,316283 |
| CDH5 | 0,343979 | 0,220381 | 1,560835 | 0,139409 | 0,367639 |
| CD70 | 0,413505 | 0,265252 | 1,558913 | 0,139862 | 0,367639 |
| Gal-4 | -0,35986 | 0,2327 | -1,54644 | 0,142833 | 0,370159 |
| TFPI | 0,236734 | 0,155213 | 1,525214 | 0,148011 | 0,378251 |
| IGF1R | 0,141278 | 0,096233 | 1,468082 | 0,162738 | 0,405192 |
| VEGF-A | 0,384948 | 0,262358 | 1,467263 | 0,162958 | 0,405192 |
| uPA | 0,268242 | 0,187136 | 1,433409 | 0,172256 | 0,415562 |
| LTBR | 0,288236 | 0,201135 | 1,433045 | 0,172358 | 0,415562 |
| CTSD | 0,257571 | 0,180426 | 1,427566 | 0,173904 | 0,415562 |
| FR-alpha | 0,325764 | 0,23178 | 1,405487 | 0,18025 | 0,424678 |
| EPHA2 | 0,3007 | 0,215036 | 1,398373 | 0,182335 | 0,424678 |
| IL-1RT1 | 0,17441 | 0,127553 | 1,367349 | 0,191657 | 0,440811 |
| TropI | 0,727104 | 0,546685 | 1,330022 | 0,20216 | 0,459228 |
| PVRL4 | 0,269139 | 0,205896 | 1,307157 | 0,210843 | 0,470844 |
| CRNN | 0,935357 | 0,718576 | 1,301681 | 0,212663 | 0,470844 |
| ITGB2 | 0,313477 | 0,243454 | 1,28762 | 0,217391 | 0,470844 |
| SPON1 | 0,197389 | 0,15334 | 1,287269 | 0,21751 | 0,470844 |
| CD163 | 0,434174 | 0,339595 | 1,278505 | 0,2205 | 0,471768 |
| CD48 | 0,302847 | 0,240909 | 1,2571 | 0,22794 | 0,479194 |
| FASLG | 0,364232 | 0,290552 | 1,253586 | 0,22918 | 0,479194 |
| vWF | 0,318797 | 0,25653 | 1,242729 | 0,233044 | 0,4818 |
| GPC1 | 0,196094 | 0,163092 | 1,202352 | 0,247864 | 0,503211 |
| PLC | 0,231353 | 0,193312 | 1,196782 | 0,249965 | 0,503211 |
| EGF | -0,4416 | 0,370324 | -1,19246 | 0,251605 | 0,503211 |
| SCGB3A2 | 0,400405 | 0,34824 | 1,149796 | 0,268231 | 0,530693 |
| IGFBP-1 | -0,58882 | 0,530148 | -1,11068 | 0,284196 | 0,552086 |
| IGFBP-2 | 0,351576 | 0,317123 | 1,108642 | 0,285044 | 0,552086 |
| TNF-R1 | 0,289422 | 0,264297 | 1,095061 | 0,290762 | 0,557294 |
| IL-18BP | 0,34727 | 0,342996 | 1,01246 | 0,327372 | 0,615039 |
| EPHB4 | 0,170857 | 0,168827 | 1,012022 | 0,327575 | 0,615039 |
| S100A11 | 0,185529 | 0,190641 | 0,973184 | 0,345895 | 0,642386 |
| NTproBNP | 0,750711 | 0,780909 | 0,961331 | 0,349123 | 0,642386 |
| PON3 | 0,308246 | 0,323105 | 0,954011 | 0,3552 | 0,647098 |
| CASP-3 | -0,33299 | 0,362468 | -0,91868 | 0,3728 | 0,660956 |
| TLT-2 | 0,176689 | 0,19264 | 0,917197 | 0,373551 | 0,660956 |
| CD207 | 0,352325 | 0,384159 | 0,917133 | 0,373584 | 0,660956 |
| IGFBP-7 | 0,148368 | 0,163506 | 0,907419 | 0,378533 | 0,661428 |
| ICAM-2 | 0,173151 | 0,19185 | 0,90253 | 0,38104 | 0,661428 |
| CCL24 | 0,333361 | 0,378689 | 0,880303 | 0,392581 | 0,667315 |
| ERBB3 | 0,120262 | 0,13737 | 0,87546 | 0,395126 | 0,667315 |
| PPY | 0,608939 | 0,701216 | 0,868404 | 0,398855 | 0,667315 |
| t-PA | 0,261417 | 0,305423 | 0,85592 | 0,405507 | 0,667315 |
| GZMH | 0,518582 | 0,608328 | 0,852471 | 0,407359 | 0,667315 |
| PECAM-1 | 0,097295 | 0,114572 | 0,849202 | 0,409118 | 0,667315 |
| RETN | 0,257134 | 0,303259 | 0,847903 | 0,409819 | 0,667315 |
| SCAMP3 | -0,45768 | 0,548826 | -0,83393 | 0,417404 | 0,673705 |
| PCSK9 | 0,151732 | 0,185278 | 0,818947 | 0,425637 | 0,679516 |
| TNFRSF4 | 0,335654 | 0,412362 | 0,813979 | 0,42839 | 0,679516 |
| CD27 | 0,181932 | 0,22758 | 0,799421 | 0,436523 | 0,686498 |
| SELE | 0,167926 | 0,216249 | 0,776538 | 0,449504 | 0,700922 |
| FGF-BP1 | -0,46012 | 0,597727 | -0,76978 | 0,453381 | 0,701026 |
| ANXA1 | 0,244719 | 0,324913 | 0,753184 | 0,462998 | 0,70993 |
| MEPE | -0,25716 | 0,349432 | -0,73595 | 0,473115 | 0,719448 |
| RSPO3 | 0,20835 | 0,286262 | 0,727829 | 0,477927 | 0,720808 |
| CA9 | 0,215895 | 0,308029 | 0,700892 | 0,4941 | 0,736607 |
| XPNPEP2 | 0,26816 | 0,38832 | 0,690566 | 0,500385 | 0,736607 |
| AZU1 | 0,290923 | 0,421308 | 0,690522 | 0,500412 | 0,736607 |
| MIC-A/B | 0,616523 | 0,924004 | 0,66723 | 0,514759 | 0,751712 |
| GRN | 0,098254 | 0,14937 | 0,65779 | 0,520641 | 0,752935 |
| FCRLB | 0,150063 | 0,2367 | 0,633979 | 0,535644 | 0,752935 |
| hK8 | 0,125651 | 0,19851 | 0,632971 | 0,536284 | 0,752935 |
| CPA1 | -0,16818 | 0,269336 | -0,62441 | 0,541742 | 0,752935 |
| FADD | -0,25077 | 0,402754 | -0,62264 | 0,542873 | 0,752935 |
| IL-17RA | -0,10734 | 0,174358 | -0,61562 | 0,547375 | 0,752935 |
| IL-6RA | 0,101296 | 0,164575 | 0,615504 | 0,547448 | 0,752935 |
| Gal-3 | 0,124025 | 0,203648 | 0,609017 | 0,551626 | 0,752935 |
| CXCL13 | 0,303935 | 0,500073 | 0,60778 | 0,552425 | 0,752935 |
| TR | 0,212323 | 0,37462 | 0,56677 | 0,579253 | 0,783402 |
| CTSZ | 0,160845 | 0,289559 | 0,555485 | 0,586753 | 0,783402 |
| CXCL16 | 0,117256 | 0,21272 | 0,55122 | 0,5896 | 0,783402 |
| JAM-A | -0,09707 | 0,177168 | -0,54792 | 0,591809 | 0,783402 |
| VIM | 0,184358 | 0,364318 | 0,506037 | 0,620186 | 0,815102 |
| HGF | 0,130378 | 0,263628 | 0,494555 | 0,628079 | 0,819621 |
| PGLYRP1 | 0,162774 | 0,334025 | 0,487311 | 0,633083 | 0,820333 |
| DLL1 | 0,103601 | 0,220503 | 0,469838 | 0,645229 | 0,830224 |
| S100A4 | 0,09911 | 0,216841 | 0,457062 | 0,654176 | 0,835891 |
| GZMB | -0,24507 | 0,54499 | -0,44968 | 0,65937 | 0,836718 |
| AR | -0,17974 | 0,429348 | -0,41864 | 0,681413 | 0,850712 |
| CHI3L1 | -0,1914 | 0,458091 | -0,41783 | 0,681994 | 0,850712 |
| MetAP 2 | 0,101613 | 0,245057 | 0,414649 | 0,684268 | 0,850712 |
| OPG | -0,08898 | 0,220701 | -0,40319 | 0,692499 | 0,855166 |
| MMP-3 | -0,12802 | 0,341677 | -0,37467 | 0,713148 | 0,874795 |
| Ep-CAM | 0,194846 | 0,560605 | 0,347564 | 0,732997 | 0,88284 |
| SELP | -0,09583 | 0,282985 | -0,33866 | 0,739562 | 0,88284 |
| TIMP4 | 0,084067 | 0,248904 | 0,33775 | 0,740232 | 0,88284 |
| TNF-R2 | 0,1314 | 0,393035 | 0,334321 | 0,742766 | 0,88284 |
| FR-gamma | -0,22911 | 0,687882 | -0,33306 | 0,743697 | 0,88284 |
| BLM hydrolase | 0,072458 | 0,225532 | 0,321276 | 0,752434 | 0,886937 |
| TGF-alpha | 0,174225 | 0,552358 | 0,31542 | 0,756788 | 0,886937 |
| CHIT1 | -0,40714 | 1,332227 | -0,30561 | 0,764102 | 0,887406 |
| DKN1A | -0,10407 | 0,34467 | -0,30195 | 0,766835 | 0,887406 |
| MPO | -0,07541 | 0,283537 | -0,26596 | 0,793892 | 0,909023 |
| FABP4 | 0,097686 | 0,370065 | 0,26397 | 0,795395 | 0,909023 |
| TFF3 | 0,139134 | 0,576074 | 0,241521 | 0,812422 | 0,920569 |
| PDGF subunit A | 0,061427 | 0,26082 | 0,235514 | 0,816995 | 0,920569 |
| hK11 | 0,038144 | 0,165192 | 0,230907 | 0,820507 | 0,920569 |
| ABL1 | -0,06981 | 0,321907 | -0,21685 | 0,831245 | 0,926964 |
| IL2-RA | -0,11943 | 0,602758 | -0,19813 | 0,845602 | 0,934461 |
| LYN | 0,029374 | 0,154982 | 0,189531 | 0,852218 | 0,934461 |
| IL-1RT2 | 0,036706 | 0,199556 | 0,183939 | 0,856525 | 0,934461 |
| SYND1 | 0,054046 | 0,323813 | 0,166904 | 0,869675 | 0,934461 |
| WISP-1 | 0,038087 | 0,230561 | 0,165191 | 0,870999 | 0,934461 |
| PRTN3 | 0,062039 | 0,380239 | 0,163158 | 0,872572 | 0,934461 |
| MK | 0,039781 | 0,245662 | 0,161936 | 0,873517 | 0,934461 |
| MUC-16 | 0,087597 | 0,588167 | 0,148932 | 0,883591 | 0,939774 |
| CSTB | 0,044742 | 0,316639 | 0,141304 | 0,88951 | 0,940631 |
| PI3 | 0,053709 | 0,438532 | 0,122474 | 0,90415 | 0,948583 |
| CPB1 | 0,025836 | 0,228485 | 0,113077 | 0,911469 | 0,948583 |
| ESM-1 | -0,02847 | 0,261928 | -0,10869 | 0,914891 | 0,948583 |
| MMP-9 | -0,03404 | 0,323746 | -0,10515 | 0,917651 | 0,948583 |
| PAI | -0,01923 | 0,195438 | -0,09838 | 0,922936 | 0,948716 |
| TXLNA | 0,023252 | 0,414729 | 0,056065 | 0,95603 | 0,977275 |
| CCL15 | 0,012623 | 0,275528 | 0,045813 | 0,964064 | 0,980042 |
| CTSV | -0,00863 | 0,262623 | -0,03286 | 0,974222 | 0,984928 |
| TNFRSF14 | 0,003792 | 0,205057 | 0,018492 | 0,98549 | 0,990875 |
| TNFRSF6B | -0,00041 | 0,397831 | -0,00103 | 0,999192 | 0,999192 |
| **Protein** | **log2FC** | **SE** | **t.value** | **p** | **q** |
| CYR61 | 1,126805 | 0,199726 | 5,641765 | 4,69E-05 | 0,008531 |
| GPNMB | 0,28544 | 0,068046 | 4,194803 | 0,000781 | 0,053475 |
| ALCAM | 0,480909 | 0,116302 | 4,135016 | 0,000881 | 0,053475 |
| ADAM 8 | 0,329935 | 0,096405 | 3,422381 | 0,00378 | 0,152379 |
| AXL | 0,693093 | 0,20748 | 3,340534 | 0,004471 | 0,152379 |
| TNFSF13B | 1,200265 | 0,365516 | 3,283753 | 0,005023 | 0,152379 |
| TNFSF13 | 0,502074 | 0,157943 | 3,178839 | 0,006228 | 0,161924 |
| PSP-D | 0,953265 | 0,321011 | 2,969569 | 0,009546 | 0,171781 |
| Gal-1 | 0,205401 | 0,069254 | 2,965931 | 0,009617 | 0,171781 |
| CEACAM5 | 1,009883 | 0,344756 | 2,929266 | 0,010361 | 0,171781 |
| FAS | 0,362698 | 0,123862 | 2,928239 | 0,010382 | 0,171781 |
| Notch 3 | 0,622769 | 0,220281 | 2,827155 | 0,012742 | 0,188084 |
| ADAM-TS 15 | 0,642813 | 0,2295 | 2,800928 | 0,013435 | 0,188084 |
| ERBB2 | 0,586779 | 0,216746 | 2,707216 | 0,016222 | 0,19149 |
| CNTN1 | 0,320786 | 0,12076 | 2,656383 | 0,017961 | 0,19149 |
| ICOSLG | 0,305613 | 0,116077 | 2,632838 | 0,018826 | 0,19149 |
| GDF-15 | 0,640398 | 0,249748 | 2,564175 | 0,021582 | 0,19149 |
| LY9 | 0,567045 | 0,221175 | 2,56378 | 0,021599 | 0,19149 |
| CXL17 | 0,616995 | 0,240845 | 2,561793 | 0,021685 | 0,19149 |
| KLK6 | 0,461895 | 0,180306 | 2,561731 | 0,021687 | 0,19149 |
| IFN-gamma-R1 | 0,337504 | 0,132233 | 2,552345 | 0,022095 | 0,19149 |
| TR-AP | 0,416906 | 0,1649 | 2,528236 | 0,023176 | 0,191727 |
| MAD homolog 5 | 0,349326 | 0,140189 | 2,491815 | 0,024905 | 0,197073 |
| PODXL | 0,202117 | 0,083149 | 2,430774 | 0,028081 | 0,212949 |
| TLR3 | 0,639419 | 0,266288 | 2,401234 | 0,029753 | 0,216603 |
| FURIN | 0,384978 | 0,161927 | 2,377485 | 0,031165 | 0,217393 |
| DLK-1 | 1,013258 | 0,440449 | 2,300511 | 0,036189 | 0,217393 |
| MMP-2 | 0,363773 | 0,158287 | 2,29819 | 0,036351 | 0,217393 |
| VEGFR-2 | 0,252485 | 0,110237 | 2,290393 | 0,036903 | 0,217393 |
| MCP-1 | 0,414949 | 0,181698 | 2,283729 | 0,03738 | 0,217393 |
| VEGFR-3 | 0,179236 | 0,078501 | 2,283237 | 0,037416 | 0,217393 |
| SCF | 0,765932 | 0,337093 | 2,272167 | 0,038223 | 0,217393 |
| SPARC | 0,210308 | 0,09729 | 2,161652 | 0,04722 | 0,259089 |
| RET | 0,512333 | 0,238447 | 2,148619 | 0,048401 | 0,259089 |
| TNFRSF19 | 0,403715 | 0,190397 | 2,120388 | 0,051056 | 0,265489 |
| SEZ6L | 0,309052 | 0,148452 | 2,081835 | 0,054897 | 0,277536 |
| U-PAR | 0,460104 | 0,225222 | 2,042891 | 0,059046 | 0,283082 |
| TCL1A | 0,722464 | 0,358146 | 2,017232 | 0,061934 | 0,283082 |
| OPN | 0,625461 | 0,311605 | 2,007221 | 0,063095 | 0,283082 |
| ERBB4 | 0,299971 | 0,14962 | 2,004884 | 0,063369 | 0,283082 |
| ITGB5 | 0,360848 | 0,180291 | 2,00147 | 0,063771 | 0,283082 |
| CD93 | 0,384191 | 0,195273 | 1,967455 | 0,067906 | 0,289831 |
| WFDC2 | 0,416317 | 0,212552 | 1,958661 | 0,069014 | 0,289831 |
| hK14 | 0,445392 | 0,228359 | 1,950404 | 0,070069 | 0,289831 |
| CD160 | 0,724578 | 0,376208 | 1,926003 | 0,073273 | 0,291423 |
| MB | 0,894677 | 0,465216 | 1,923143 | 0,073656 | 0,291423 |
| TNFSF10 | 0,329902 | 0,17696 | 1,864272 | 0,081973 | 0,301616 |
| KLK13 | 0,581421 | 0,316727 | 1,835718 | 0,086302 | 0,301616 |
| MSLN | 0,801091 | 0,437848 | 1,82961 | 0,087254 | 0,301616 |
| TFPI-2 | 0,378516 | 0,206957 | 1,828955 | 0,087357 | 0,301616 |
| CCL16 | 0,440525 | 0,241444 | 1,82454 | 0,088051 | 0,301616 |
| 5'-NT | 0,431977 | 0,237934 | 1,815536 | 0,089483 | 0,301616 |
| ST2 | 0,556579 | 0,307409 | 1,810552 | 0,090284 | 0,301616 |
| EGFR | 0,240946 | 0,135126 | 1,783128 | 0,094809 | 0,301616 |
| CEACAM1 | 0,109528 | 0,061903 | 1,769349 | 0,097157 | 0,301616 |
| ITGAV | 0,301924 | 0,171434 | 1,761164 | 0,098576 | 0,301616 |
| SHPS-1 | 0,395713 | 0,224798 | 1,760308 | 0,098725 | 0,301616 |
| AP-N | 0,221825 | 0,126481 | 1,753813 | 0,099865 | 0,301616 |
| MIA | 0,13787 | 0,078633 | 1,75334 | 0,099949 | 0,301616 |
| RARRES2 | 0,155899 | 0,089192 | 1,7479 | 0,100914 | 0,301616 |
| COL1A1 | 0,409303 | 0,234302 | 1,746904 | 0,101091 | 0,301616 |
| CPE | 0,358141 | 0,210134 | 1,704342 | 0,108937 | 0,309103 |
| TNFRSF10C | 0,386037 | 0,227169 | 1,699337 | 0,109893 | 0,309103 |
| IL-6 | 0,842478 | 0,496493 | 1,696859 | 0,11037 | 0,309103 |
| WIF-1 | 0,319613 | 0,189576 | 1,685934 | 0,112491 | 0,309103 |
| LDL receptor | 0,477171 | 0,283829 | 1,681195 | 0,113423 | 0,309103 |
| TGFR-2 | 0,285966 | 0,170286 | 1,67933 | 0,113791 | 0,309103 |
| LYPD3 | 0,526076 | 0,316179 | 1,663854 | 0,116887 | 0,312845 |
| CDH5 | 0,343979 | 0,220381 | 1,560835 | 0,139409 | 0,363642 |
| CD70 | 0,413505 | 0,265252 | 1,558913 | 0,139862 | 0,363642 |
| Gal-4 | -0,35986 | 0,2327 | -1,54644 | 0,142833 | 0,366136 |
| TFPI | 0,236734 | 0,155213 | 1,525214 | 0,148011 | 0,37414 |
| IGF1R | 0,141278 | 0,096233 | 1,468082 | 0,162738 | 0,400787 |
| VEGF-A | 0,384948 | 0,262358 | 1,467263 | 0,162958 | 0,400787 |
| uPA | 0,268242 | 0,187136 | 1,433409 | 0,172256 | 0,411045 |
| LTBR | 0,288236 | 0,201135 | 1,433045 | 0,172358 | 0,411045 |
| CTSD | 0,257571 | 0,180426 | 1,427566 | 0,173904 | 0,411045 |
| FR-alpha | 0,325764 | 0,23178 | 1,405487 | 0,18025 | 0,420062 |
| EPHA2 | 0,3007 | 0,215036 | 1,398373 | 0,182335 | 0,420062 |
| IL-1RT1 | 0,17441 | 0,127553 | 1,367349 | 0,191657 | 0,43602 |
| PVRL4 | 0,269139 | 0,205896 | 1,307157 | 0,210843 | 0,471271 |
| CRNN | 0,935357 | 0,718576 | 1,301681 | 0,212663 | 0,471271 |
| ITGB2 | 0,313477 | 0,243454 | 1,28762 | 0,217391 | 0,471271 |
| SPON1 | 0,197389 | 0,15334 | 1,287269 | 0,21751 | 0,471271 |
| CD163 | 0,434174 | 0,339595 | 1,278505 | 0,2205 | 0,47213 |
| CD48 | 0,302847 | 0,240909 | 1,2571 | 0,22794 | 0,479433 |
| FASLG | 0,364232 | 0,290552 | 1,253586 | 0,22918 | 0,479433 |
| vWF | 0,318797 | 0,25653 | 1,242729 | 0,233044 | 0,481978 |
| GPC1 | 0,196094 | 0,163092 | 1,202352 | 0,247864 | 0,503211 |
| PLC | 0,231353 | 0,193312 | 1,196782 | 0,249965 | 0,503211 |
| EGF | -0,4416 | 0,370324 | -1,19246 | 0,251605 | 0,503211 |
| SCGB3A2 | 0,400405 | 0,34824 | 1,149796 | 0,268231 | 0,53063 |
| IGFBP-1 | -0,58882 | 0,530148 | -1,11068 | 0,284196 | 0,551894 |
| IGFBP-2 | 0,351576 | 0,317123 | 1,108642 | 0,285044 | 0,551894 |
| TNF-R1 | 0,289422 | 0,264297 | 1,095061 | 0,290762 | 0,557039 |
| IL-18BP | 0,34727 | 0,342996 | 1,01246 | 0,327372 | 0,614625 |
| EPHB4 | 0,170857 | 0,168827 | 1,012022 | 0,327575 | 0,614625 |
| S100A11 | 0,185529 | 0,190641 | 0,973184 | 0,345895 | 0,642377 |
| PON3 | 0,308246 | 0,323105 | 0,954011 | 0,3552 | 0,652995 |
| CASP-3 | -0,33299 | 0,362468 | -0,91868 | 0,3728 | 0,666591 |
| TLT-2 | 0,176689 | 0,19264 | 0,917197 | 0,373551 | 0,666591 |
| CD207 | 0,352325 | 0,384159 | 0,917133 | 0,373584 | 0,666591 |
| IGFBP-7 | 0,148368 | 0,163506 | 0,907419 | 0,378533 | 0,66682 |
| ICAM-2 | 0,173151 | 0,19185 | 0,90253 | 0,38104 | 0,66682 |
| CCL24 | 0,333361 | 0,378689 | 0,880303 | 0,392581 | 0,671955 |
| ERBB3 | 0,120262 | 0,13737 | 0,87546 | 0,395126 | 0,671955 |
| PPY | 0,608939 | 0,701216 | 0,868404 | 0,398855 | 0,671955 |
| t-PA | 0,261417 | 0,305423 | 0,85592 | 0,405507 | 0,671955 |
| GZMH | 0,518582 | 0,608328 | 0,852471 | 0,407359 | 0,671955 |
| PECAM-1 | 0,097295 | 0,114572 | 0,849202 | 0,409118 | 0,671955 |
| RETN | 0,257134 | 0,303259 | 0,847903 | 0,409819 | 0,671955 |
| SCAMP3 | -0,45768 | 0,548826 | -0,83393 | 0,417404 | 0,678282 |
| PCSK9 | 0,151732 | 0,185278 | 0,818947 | 0,425637 | 0,683922 |
| TNFRSF4 | 0,335654 | 0,412362 | 0,813979 | 0,42839 | 0,683922 |
| CD27 | 0,181932 | 0,22758 | 0,799421 | 0,436523 | 0,690846 |
| SELE | 0,167926 | 0,216249 | 0,776538 | 0,449504 | 0,705256 |
| FGF-BP1 | -0,46012 | 0,597727 | -0,76978 | 0,453381 | 0,70526 |
| ANXA1 | 0,244719 | 0,324913 | 0,753184 | 0,462998 | 0,714115 |
| MEPE | -0,25716 | 0,349432 | -0,73595 | 0,473115 | 0,723588 |
| RSPO3 | 0,20835 | 0,286262 | 0,727829 | 0,477927 | 0,724856 |
| CA9 | 0,215895 | 0,308029 | 0,700892 | 0,4941 | 0,740448 |
| XPNPEP2 | 0,26816 | 0,38832 | 0,690566 | 0,500385 | 0,740448 |
| AZU1 | 0,290923 | 0,421308 | 0,690522 | 0,500412 | 0,740448 |
| MIC-A/B | 0,616523 | 0,924004 | 0,66723 | 0,514759 | 0,755534 |
| GRN | 0,098254 | 0,14937 | 0,65779 | 0,520641 | 0,75595 |
| FCRLB | 0,150063 | 0,2367 | 0,633979 | 0,535644 | 0,75595 |
| hK8 | 0,125651 | 0,19851 | 0,632971 | 0,536284 | 0,75595 |
| CPA1 | -0,16818 | 0,269336 | -0,62441 | 0,541742 | 0,75595 |
| FADD | -0,25077 | 0,402754 | -0,62264 | 0,542873 | 0,75595 |
| IL-17RA | -0,10734 | 0,174358 | -0,61562 | 0,547375 | 0,75595 |
| IL-6RA | 0,101296 | 0,164575 | 0,615504 | 0,547448 | 0,75595 |
| Gal-3 | 0,124025 | 0,203648 | 0,609017 | 0,551626 | 0,75595 |
| CXCL13 | 0,303935 | 0,500073 | 0,60778 | 0,552425 | 0,75595 |
| TR | 0,212323 | 0,37462 | 0,56677 | 0,579253 | 0,786199 |
| CTSZ | 0,160845 | 0,289559 | 0,555485 | 0,586753 | 0,786199 |
| CXCL16 | 0,117256 | 0,21272 | 0,55122 | 0,5896 | 0,786199 |
| JAM-A | -0,09707 | 0,177168 | -0,54792 | 0,591809 | 0,786199 |
| VIM | 0,184358 | 0,364318 | 0,506037 | 0,620186 | 0,817927 |
| HGF | 0,130378 | 0,263628 | 0,494555 | 0,628079 | 0,822377 |
| PGLYRP1 | 0,162774 | 0,334025 | 0,487311 | 0,633083 | 0,823008 |
| DLL1 | 0,103601 | 0,220503 | 0,469838 | 0,645229 | 0,832848 |
| S100A4 | 0,09911 | 0,216841 | 0,457062 | 0,654176 | 0,838451 |
| GZMB | -0,24507 | 0,54499 | -0,44968 | 0,65937 | 0,839198 |
| AR | -0,17974 | 0,429348 | -0,41864 | 0,681413 | 0,852992 |
| CHI3L1 | -0,1914 | 0,458091 | -0,41783 | 0,681994 | 0,852992 |
| MetAP 2 | 0,101613 | 0,245057 | 0,414649 | 0,684268 | 0,852992 |
| OPG | -0,08898 | 0,220701 | -0,40319 | 0,692499 | 0,857379 |
| MMP-3 | -0,12802 | 0,341677 | -0,37467 | 0,713148 | 0,876979 |
| Ep-CAM | 0,194846 | 0,560605 | 0,347564 | 0,732997 | 0,884659 |
| SELP | -0,09583 | 0,282985 | -0,33866 | 0,739562 | 0,884659 |
| TIMP4 | 0,084067 | 0,248904 | 0,33775 | 0,740232 | 0,884659 |
| TNF-R2 | 0,1314 | 0,393035 | 0,334321 | 0,742766 | 0,884659 |
| FR-gamma | -0,22911 | 0,687882 | -0,33306 | 0,743697 | 0,884659 |
| BLM hydrolase | 0,072458 | 0,225532 | 0,321276 | 0,752434 | 0,888616 |
| TGF-alpha | 0,174225 | 0,552358 | 0,31542 | 0,756788 | 0,888616 |
| CHIT1 | -0,40714 | 1,332227 | -0,30561 | 0,764102 | 0,888942 |
| DKN1A | -0,10407 | 0,34467 | -0,30195 | 0,766835 | 0,888942 |
| MPO | -0,07541 | 0,283537 | -0,26596 | 0,793892 | 0,910452 |
| FABP4 | 0,097686 | 0,370065 | 0,26397 | 0,795395 | 0,910452 |
| TFF3 | 0,139134 | 0,576074 | 0,241521 | 0,812422 | 0,921804 |
| PDGF subunit A | 0,061427 | 0,26082 | 0,235514 | 0,816995 | 0,921804 |
| hK11 | 0,038144 | 0,165192 | 0,230907 | 0,820507 | 0,921804 |
| ABL1 | -0,06981 | 0,321907 | -0,21685 | 0,831245 | 0,928138 |
| IL2-RA | -0,11943 | 0,602758 | -0,19813 | 0,845602 | 0,935177 |
| LYN | 0,029374 | 0,154982 | 0,189531 | 0,852218 | 0,935177 |
| IL-1RT2 | 0,036706 | 0,199556 | 0,183939 | 0,856525 | 0,935177 |
| SYND1 | 0,054046 | 0,323813 | 0,166904 | 0,869675 | 0,935177 |
| WISP-1 | 0,038087 | 0,230561 | 0,165191 | 0,870999 | 0,935177 |
| PRTN3 | 0,062039 | 0,380239 | 0,163158 | 0,872572 | 0,935177 |
| MK | 0,039781 | 0,245662 | 0,161936 | 0,873517 | 0,935177 |
| MUC-16 | 0,087597 | 0,588167 | 0,148932 | 0,883591 | 0,940431 |
| CSTB | 0,044742 | 0,316639 | 0,141304 | 0,88951 | 0,941226 |
| PI3 | 0,053709 | 0,438532 | 0,122474 | 0,90415 | 0,948934 |
| CPB1 | 0,025836 | 0,228485 | 0,113077 | 0,911469 | 0,948934 |
| ESM-1 | -0,02847 | 0,261928 | -0,10869 | 0,914891 | 0,948934 |
| MMP-9 | -0,03404 | 0,323746 | -0,10515 | 0,917651 | 0,948934 |
| PAI | -0,01923 | 0,195438 | -0,09838 | 0,922936 | 0,949008 |
| TXLNA | 0,023252 | 0,414729 | 0,056065 | 0,95603 | 0,977514 |
| CCL15 | 0,012623 | 0,275528 | 0,045813 | 0,964064 | 0,980221 |
| CTSV | -0,00863 | 0,262623 | -0,03286 | 0,974222 | 0,985047 |
| TNFRSF14 | 0,003792 | 0,205057 | 0,018492 | 0,98549 | 0,990935 |
| TNFRSF6B | -0,00041 | 0,397831 | -0,00103 | 0,999192 | 0,999192 |
